# Utilizing machine learning in predicting yields of products in biomass thermochemical conversion processes

**DOI:** 10.1186/s40643-025-00956-8

**Published:** 2025-11-06

**Authors:** Kareem H. Hamad, M. Hanafy, A. Wafiq

**Affiliations:** 1 Department of Chemical Engineering, Egyptian Academy for Engineering and Advanced Technology, Cairo Belbes Street, Giza, 3056 Egypt; 2 Department of Chemical Engineering, Cairo University, Giza, 12613 Egypt

**Keywords:** Bioenergy, Gasification, Machine learning, Pyrolysis, Waste management

## Abstract

Agricultural and municipal waste management is one of the most important topics nowadays due to the high rate of waste generation. Bioenergy is one of the key technologies to achieve the global net zero CO_2_ emissions goal. In the era of biorefineries, and especially for new types of waste and new waste blends, a feasibility study is required to identify the optimum waste conversion process which is usually preceded by experimental investigation. Especially for developing countries, securing the corresponding funds is usually challenging. In this research work, machine learning has been applied to develop a statistical model based on 144 samples of the published experimental data for different types of wastes undergoing three thermochemical conversion processes; namely, slow pyrolysis, fast pyrolysis and gasification. Statistical analysis has been performed and models were built with (95% confidence level) which correlate the various products’ yields with the waste composition and operating conditions. These models provide a guide to researchers regarding the expected yield of products for each of the three studied processes so as to discard the non-promising processes for the used type of waste. This will accordingly minimize the number of required experimental runs; hence saving time and money. A decision matrix was also developed based on the statistical models of each of the studied thermochemical processes. It was concluded that when carbon content is moderate (40–46%, by mass), gasification process is favored if the hydrogen content is high; otherwise, slow pyrolysis is favored. On the other hand, if the waste has high carbon content (above 47%) then fast pyrolysis is favored.

**Graphical abstract:**

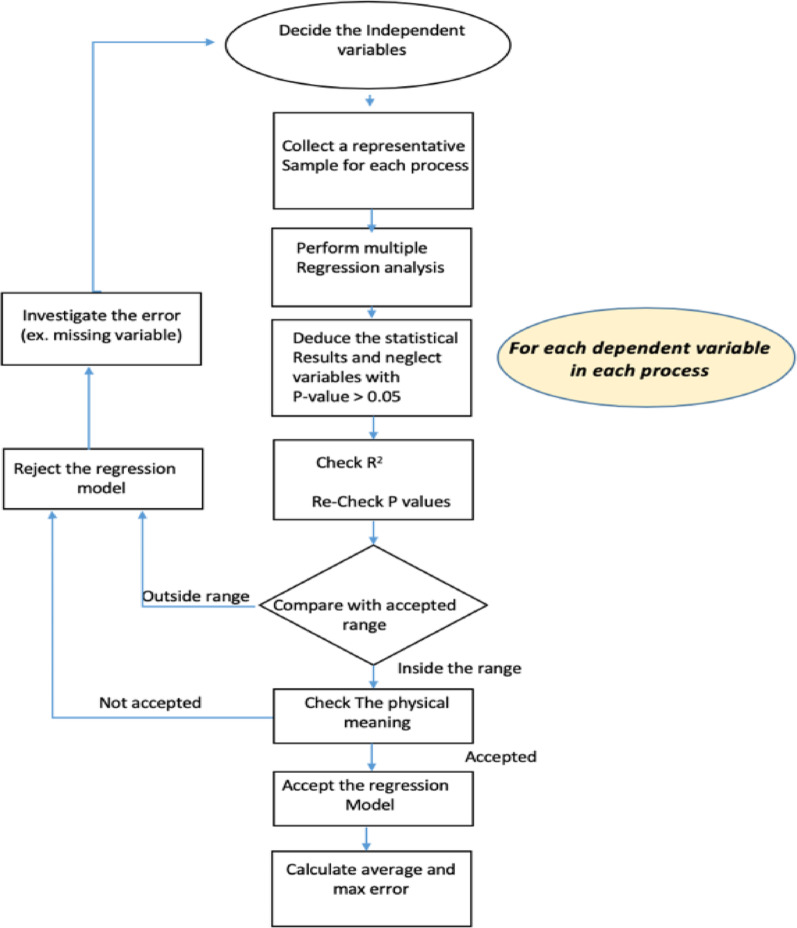

**Supplementary Information:**

The online version contains supplementary material available at 10.1186/s40643-025-00956-8.

## Introduction

With the gradual increase in global population, depletion of fossil fuel sources, and the increased global sustainability and net zero CO_2_ emissions target, the valorization of alternative sources of energy should be maximized. According to the Sixth Assessment Report of the Intergovernmental Panel on Climate Change (IPCC), bioenergy is an important pathway to climate change mitigation, and its association with carbon capture and storage is inevitable to achieve the net zero CO_2_ emissions target (IPCC 2022). One of the key bioenergy routes is the waste to energy (WTE) processes (Yang et al. 2006; Ibikunle et al. 2020; Kumar et al. 2019; Javed et al. 2016; Langsdorf et al. 2021; Naqvi et al. 2020; Kumar and Samadder 2017). WTE processes provide solution to an additional major global problem related to the mismanagement of the agricultural and municipal solid wastes which are generated at high rate in many countries. Mismanagement of solid wastes (e.g. open burning) causes serious air, water and soil pollution in addition to retarding economic sustainability (Ferronato and Torretta 2019; Souza et al. 2017; Ferronato et al. 2018; Goel 2017). As of 2016 statistics, it is estimated that the world generated 2.01 billion tons of municipal solid wastes; the value which is expected to reach about 3.4 billion tons in 2050. On the other hand, the global agricultural wastes were estimated to be more than four and half times that of municipal solid wastes (Kaza et al. 2018; Wang et al. 2022; D. Hoornweg PBT 2012), the fact which reflects the necessity of their effective utilization especially that a lot of them were reported to have good potential as energy resource (Bentsen et al. 2014; Kalyani and Pandey 2014; Titiloye et al. 2013; Silva Herran and Nakata 2012).

The conversion of biomass generally and waste specifically to liquid and gaseous forms of energy usually takes place through two broad pathways; biochemical and thermochemical (García-Velásquez and Cardona 2019). Unlike the biochemical route, the thermochemical route does not need bacteria or enzymes as the biomass conversion takes place as a result of applying high temperatures enough to dissociate the biomass molecules into liquid and gaseous energy forms. The most famous processes under the thermochemical conversion route are incineration, pyrolysis and gasification (Basu 2010). Incineration is the most widely used WTE processes; however, it is criticized for its environmental performance as it emits higher loads of several pollutants including nitrogen oxides and particulate matter in addition to being less energy efficient compared to other thermochemical WTE processes such as pyrolysis and gasification (Zaman 2013; Astrup et al. 2015; Evangelisti et al. 2015; Vaish et al. 2017). Pyrolysis and gasification are two more environmentally-friendly thermochemical processes that are also commercial and getting a lot of attention in the literature (Dong et al. 2019; Robazza and Neumann 2024; Lin et al. 2016; Wang et al. 2020; Ashoor et al. 2023). Hence, this research focused on the pyrolysis and the gasification thermochemical processes.

Pyrolysis is the thermal degradation of the solid feedstock in the absence of oxygen. During pyrolysis, large hydrocarbon molecules break down into smaller molecules of gas, liquid and char. Pyrolysis can be generally classified into slow pyrolysis and fast pyrolysis according to the applied heating rate (Basu 2010). In fast pyrolysis high heating rates and short residence time are applied, and the pyrolysis temperature is between 500 and 650 °C thus generating bio-oil as a main product (Basu 2010; Fernandez et al. 2022; Diebold and Bridgwater 2017). Bio-oil can be used as a fuel or as a precursor to produce gasoline, diesel or other chemicals. For slow pyrolysis low heating rates and long residence time are applied, and the pyrolysis temperature is about 400 °C thus generating char as a main product. Char can be used in adsorption applications and as a fuel source (Basu 2010; Huber and Corma 2007; Williams and Besler 1996). On the other hand, gasification is the process in which the solid feedstock is converted into a gaseous product usually called syngas (mainly carbon monoxide and hydrogen) in low-oxygen conditions (Basu 2010; Waheed et al. 2022). Syngas produced from the gasification process can be used in gas turbines for electricity generation, and can be also used to produce gasoline, and methanol (Basu 2010).

Techno-economic feasibility assessment studies are crucial to decide whether the utilization of one of the above-mentioned thermochemical conversion processes is suitable for a specific waste feedstock. One of the most important inputs to such feasibility studies is the yield of each product from such processes. In the literature, there are two key methods to identify such product yields; the first method is to conduct the corresponding experiments (Vera et al. 2014; Ortiz-Sanchez et al. 2020; Matrapazi and Zabaniotou 2020; Xia et al. 2018; Shen et al. 2017), while the second method is to rely on thermodynamic process simulators (e.g. ASPEN Plus) or different machine learning approaches such as Linear and Nonlinear correlations, Artificial neural networks (ANN), Support vector machine (SVM), and Random Forest (RF) (Astrup et al. 2015; Anex et al. 2010; Bora et al. 2020; AlNouss et al. 2020; Andiappan et al. 2015; Jana and De 2015; Bing et al. 2018; Wafiq and Hanafy 2015; Im-orb et al. 2018; Djandja et al. 2021; Elmaz et al. 2020; Xing et al. 2019). Machine learning is a branch that uses data to predict accurate outputs, and it has two main categories; supervised learning and unsupervised learning. Supervised learning depends on given inputs and outputs to make prediction such as regression and classification. Unsupervised learning analyze data without labeling, where it depends on similarities and patterns such as clustering, association and dimensionality reduction (Delua 2021). The first method (i.e. conducting experiments) is more accurate but requires prior installation of experimental setup for each of the processes; hence it is both time and cost intensive option. On the other hand, the second way is less time consuming; however, it is less accurate (Lee et al. 2007; Lopez-Urionabarrenechea et al. 2012).

For the optimum design of a biorefinery and biomass conversion parks, usually a high-level estimate for the projected yields of different processes is required (Van Dael et al. 2013a, 2013b; Tay et al. 2012). Biochemical Methane Potential (BMP) indicator is a famous example that is used to predict the methane yield in anaerobic digestion. BMP can be used to predict the amount of methane that can be produced from one gram of organic substrates (Filer et al. 2019) using both experimental and theoretical models (Jingura and Kamusoko 2017). Using theoretical methods can quickly guide the researchers about the expected biogas yield from anaerobic digestion; hence, giving good inputs for feasibility studies. To the authors’ knowledge, similar indicators for slow pyrolysis, fast pyrolysis, and gasification thermochemical processes are scarce in the literature. Lopez et al. has applied empiric modeling to predict the yields of the different pyrolysis products as a function of the input waste composition for packaging waste samples (Lopez-Urionabarrenechea et al. 2012). Dellavedova et al. (Dellavedova et al. 2012) and Gil et al. (Gil et al. 2019) have applied multivariate statistical techniques to draw some conclusions regarding the effect of biomass properties on gasification yields; however, without equations.

In this research work, empirical statistical models have been developed based on published experimental data for different types of wastes for slow pyrolysis, fast pyrolysis and gasification. Statistical analysis has been performed to reach an equation (with a 95% confidence level, and low percentage of error) which correlates the yields of solid, liquid and gaseous products for each of the three studied processes with the waste composition and operating conditions. The developed models provide a guide to researchers regarding the expected yield of products for each of the three studied processes so as to discard the non-promising processes for the used type of waste and accordingly minimize the number of required experimental runs. On the other hand, this research work provides a reliable rapid assessment inputs to the feasibility studies that are used to select the best waste to energy thermochemical conversion process on case-by-case basis.

## Methods

### Applied statistical modeling techniques

This research work is based on a supervised machine learning method; namely, regression analysis. The latter is one of the most common and comprehensive statistical and machine learning algorithms (Maulud and Abdulazeez 2020). Regression analysis is simply a statistical technique that aims to model the relationship between variables. Regression is usually an approximation to the true functional relationship between the variables of concern. Such true functional relationships are often based on scientific theory and usually named as “mechanistic models”, while the regression models are usually referred to as “empirical models”.

Regression models have various forms; the most famous of which are simple linear regression models, multiple linear regression models, polynomial regression models, and nonlinear regression models. A simple linear regression model is a model with a single independent variable x (also known as regressor) that has a straight-line relationship with a dependent variable y (also known as response). A regression model where the straight-line relationship involves more than one independent variable is called a multiple linear regression model. When the relationship between variables is curvilinear, polynomial regression models can be employed. In some situations, the true relationship between independent and dependent variables can be more complex (e.g. a differential equation or the solution to a differential equation); hence, in this case non-linear regression models need to be employed.

Usually, the starting point in regression analysis is to assume a linear relationship between the variables. While linear regression analysis has some advantages such as being easy to apply by users via utilizing available computational packages, using moderate sample size, and being able to evaluate the output models via interpreting their physical meanings, it still has some other drawbacks such as assuming the linearity of the variables. However, a nonlinear relationship can be identified upon testing the resulting linear regression model. One way to test that is by using residual plots which will be explained below. In some cases, a nonlinear function can be linearized by using a suitable transformation (e.g. by using logarithms for exponential or power-law relationships) (Montgomery et al. 2021; Neter et al. 2005). Hence, the modeling strategy followed in this research work is to:

Identify the independent variables which are thought to be affecting the dependent variables (i.e. the product yields in the studied thermochemical conversion technologies)

1. Use multiple linear regression to develop the model
2. Test the generated multiple linear regression model using various methods (will be explained in details below)
3. Identify whether any non-linearity is suspected. If so, use the necessary transformation to linearize the model
4. In case non-linearity is proven, and it is not possible to use transformation to linearize the model, employ a non-linear regression model.

As will be shown in the results, the resulting regression equations were well-fitted via the multiple linear regression models, and there was no need for employing non-linear regression models.

Multiple linear regression analysis was used to relate several independent variables to a dependent variable (Maulud and Abdulazeez 2020; Guo et al. 2019). The general output of the multiple linear regression analysis is according to Eq. 1.

1 $$ Y = \, a \, + \, b_{1} X_{1} + \, b_{2} X_{2} + \, \ldots . + \, b_{n} X_{n} $$

This equation relates the dependent variable (Y) to the independent variables (X_1_, X_2_ and X_n_). The.

intercept on the Y axis is (a), while b_1_, b_2_ and b_n_ are the regression coefficients.

Several statistical tests have been applied to evaluate the reliability and significance of the generated regression equations. The first applied test is the* P* value, which was used to determine the confidence level of the developed model and to reject the null hypothesis that the results are obtained randomly and that the correlation is not correct. For 95% confidence level, the null hypothesis is rejected when *P *value for all independent variables is below 5%. The second applied test is the Coefficient of Determination (R Square) which was used to reflect the variance of one variable with respect to the other. The adjusted coefficient of determination (Adjusted R Square) shows the same as the coefficient of determination; however, it takes into consideration the variables used in the equation. As long as strong related variables are used, the value of it is increased and vice versa. Equations with coefficient of determination values higher than 0.7 were considered to be statistically significant (Henseler et al. 2009). The third applied test is the residuals plot. Residuals are the difference between the real value of the dependent variable and the value of the calculated variable, where as long as the residuals are dispersed around the axis uniformly, the linear regression model can be accepted. The final applied test is the normal probability plot. It was used to confirm that the points are normally distributed, which is the case when the data is represented in a nearly straight line (Zwillinger 2002).

### Work procedure for regression models development

**Fig. 1:**
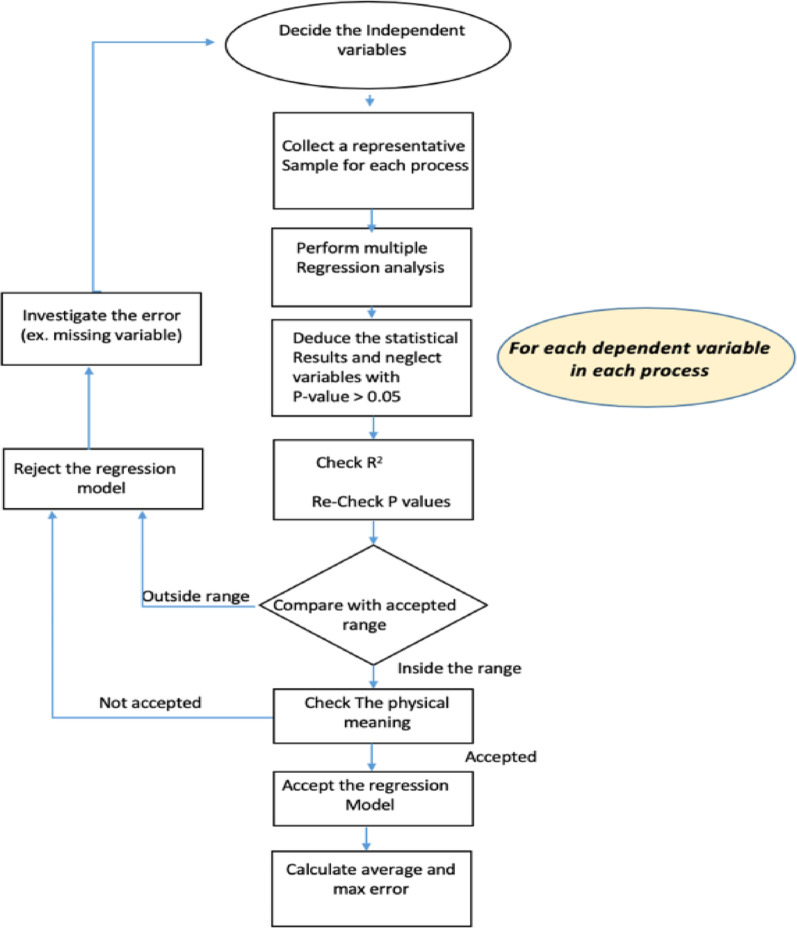
Work procedure for regression models development

Figure 1 presents the procedure followed in this research work to develop the model equations for each of the three studied thermochemical processes. For each process, first of all the independent variables which are projected to have significant effect on the yields of the different products were decided. Based on that, a representative sample of experimental results was collected from literature while making sure that all the suspected independent variables are reported. A multiple regression analysis was then performed for each product yield, and the resulting regression model equation was assessed statistically (based on the previously mentioned tests). If the model equation passes the assessment, its physical meaning was assessed to decide whether it is considered as a representative model and the corresponding error is calculated. On the other hand, if it is not compatible physically or statistically, then the regression model was rejected, and investigation of the error was made (for example missing independent variable that can have significant impact on the yield).

The error was calculated using the following formulas:

2 $$ \begin{aligned} & Minimum/MaximumError \\ & = \frac{{Minimum/Maximum \; absolute \; residual}}{{Corresponding \; actual \; yield \; from \; expirements}} \\ \end{aligned} $$

3 $$ Average Error = \frac{Average \; absolute \; residual}{{Average \; yield \; from \; expirements}} $$

Some of the processes (i.e. slow pyrolysis, fast pyrolysis, gasification) has two accepted models; each model has some variables taken into consideration (ultimate analysis only, proximate analysis only, or both). When a process has two models, a discussion was made to decide which model is better regarding the physical meaning and the statistical significance.

### Variables considered in statistical analysis for each process

#### Slow pyrolysis

The independent variables taken into consideration for the slow pyrolysis were, the ultimate analysis of the waste (C, H, O, N, S), proximate analysis of the waste; namely, fixed carbon (FC), volatile matter (VM), ash and moisture, in addition to the pyrolysis temperature (^o^C), average particle size, and heating rate. On the other hand, the dependent variables assessed were gas yield (wt%), oil yield (wt%), and char yield (wt%).

#### Fast pyrolysis

The independent variables taken into consideration for the fast pyrolysis were the ultimate analysis of the waste, proximate analysis of the waste, pyrolysis temperature (^o^C), and average particle size. The dependent variables assessed were gas yield (wt%), oil yield (wt%), and char yield (wt%).

#### Gasification

The independent variables taken into consideration for the gasification process were the ultimate analysis of the waste, proximate analysis of the waste, gasification temperature (^o^C), and equivalence ratio (ER) which is the ratio between the air used for gasification and stoichiometric air required for complete combustion. On the other hand, the dependent variables assessed were hydrogen yield (m^3^/kg of feed), carbon monoxide yield (m^3^/kg of feed), carbon dioxide yield (m^3^/kg of feed), and methane yield (m^3^/kg of feed). It is to be highlighted that the research scope was for air gasification.

#### Statistical modeling

To develop a regression model for each of the three thermochemical processes, 144 experimental results were collected from several published research papers. The number of experiments used in each model exceeded the minimum theoretical number of experiments for a linear regression equation which is equal to 2^k^ (Dean et al. 2017), where k is the number of variables. The selected papers included experiments previously done by other researchers and published by reputable academic publishers, accessed via platforms such as Elsevier and Springer nature. Google Scholar was the search engine mainly used with the main keywords being biomass, slow pyrolysis, gasification, and fast pyrolysis. Three criteria were taken into consideration to select the papers used in this research work. The first one was to find papers published in a reputable source, the second one was to make sure that the raw material used is biomass, and the last one was to make sure of the availability and clarity of all the information required to develop the models; including but not limited to the proximate analysis, ultimate analysis, particle size, heating rates, and operating conditions. Several regression models were accordingly developed, from which some models were not accepted either due to high error, or being statistically/physically not significant. As shown in the Results and Discussion, for each of the studied thermochemical processes, a regression model was developed for each of the main products (e.g. char, bio-oil, and gas for the slow pyrolysis). The final experiments from which the calculated models were built is shown in supplementary Tables S1–S3).

### Ranges of regression models’ applicability

The ranges of each model applicability depend on the minimum and maximum values of each independent variable used to develop the models. Hence, to use such model equations, the independent variables for each of the thermochemical processes need to be within the ranges shown in Table 1.

**Table 1:** Range of regression models’ applicability for the studied thermochemical processes

Variables	Fast pyrolysis		Slow pyrolysis		Gasification	
	Min	Max	Min	Max	Min	Max
N (% dry basis)	0.10	5.50	0.10	4.00	0.10	9.10
S (% dry basis)	0	0.97	0	1.00	0.00	2.00
H (% dry basis)	5.00	7.50	4.00	8.30	4.90	8.50
O (% dry basis)	31.40	56.10	38.10	54.10	27.60	48.50
C (% dry basis)	38.10	58.50	39.50	50.90	42.80	59.20
Temperature (℃)	400	1000	350	600	450	932
Ash (% dry basis)	1.00	23.60	0.55	14.00	0.40	10.00
FC (% dry basis)	12.20	24.20	6.50	22.00	0	20.40
VM dry (% dry basis)	56.20	82.60	68.30	90.00	0	83.00
Particle size (mm)	0.08	2.00	0.25	3.20		
Heating rate			5.00	50.00		
ER					0.15	0.40

### Work procedure for feasibility assessment

**Fig. 2:**
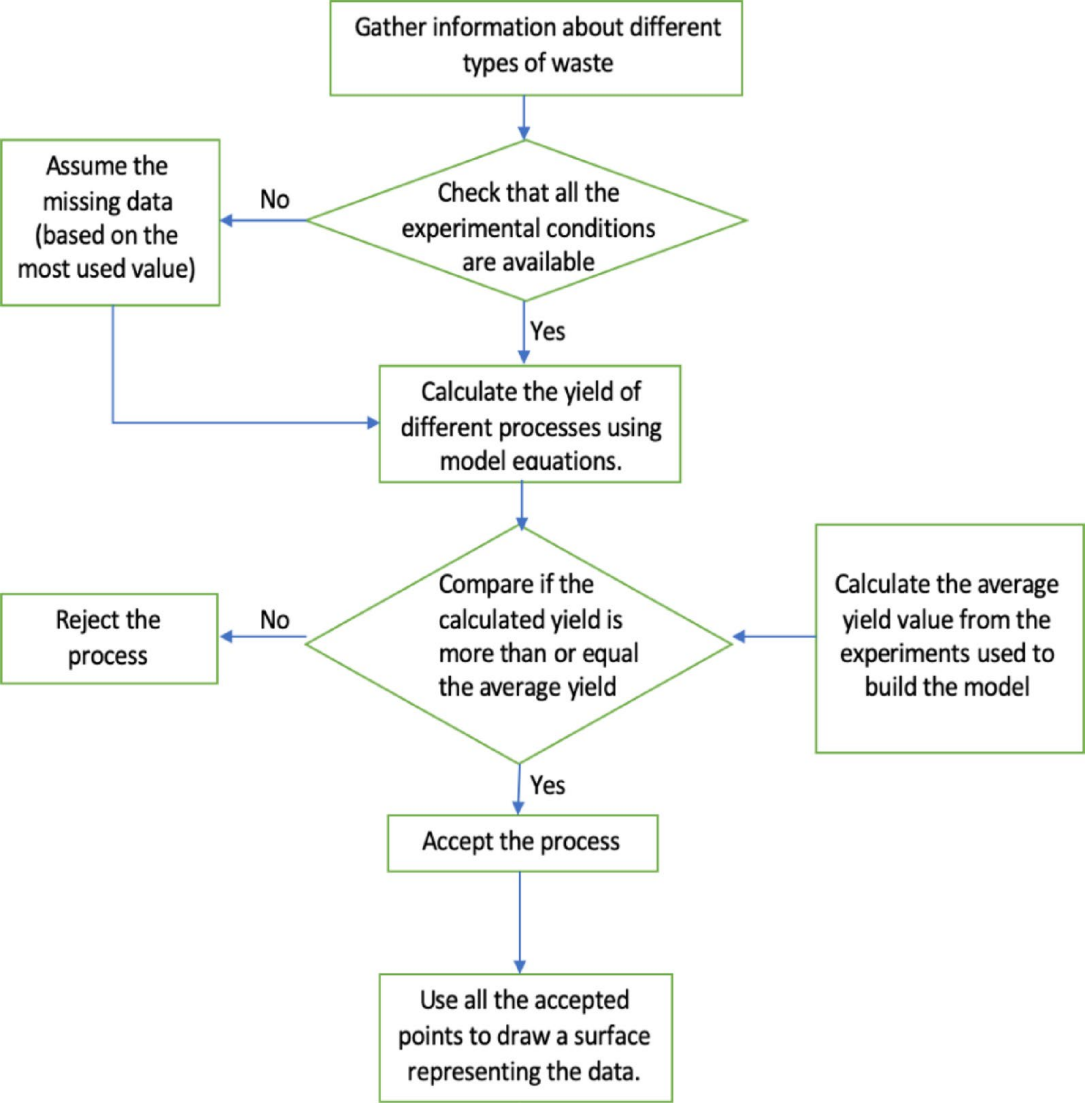
Work procedure for feasibility assessment

In this research as well, a work procedure has been followed to assess the feasibility of each thermochemical process for a specific waste type as shown in Fig. 2. The feasibility is based on the yield of the main product of each of the three studied thermochemical processes; namely, the bio-char yield for slow pyrolysis, the bio-oil yield for fast pyrolysis, and the calorific values of the resulting gases for gasification. The first step for feasibility assessment of a specific waste type is to calculate the product yields for each of the thermochemical processes via the developed statistical models. For each process, a criterion was made to decide whether the feedstock is promising for this process. In the case of slow pyrolysis, the bio-char yield for each waste type was compared to the average value obtained by the model; which is equivalent to 34%. In the case of fast pyrolysis, the yield of bio-oil was compared to the average value obtained by the model; which is equivalent to 31%. Finally, in the case of gasification, the calorific values from the resulting gases were compared to the average calorific value; which is 6 MJ/kg feed. A surface plot was then developed using MATLAB to decide the best management technique for each type of waste knowing only the carbon, hydrogen, and oxygen contents.

## Results and discussion

For each process, a regression model was deduced taking into consideration all the assessed variables (listed above in the “Methods” section) as illustrated in the following sections.

### Fast pyrolysis model

#### Gas yield regression model

**Fig. 3:**
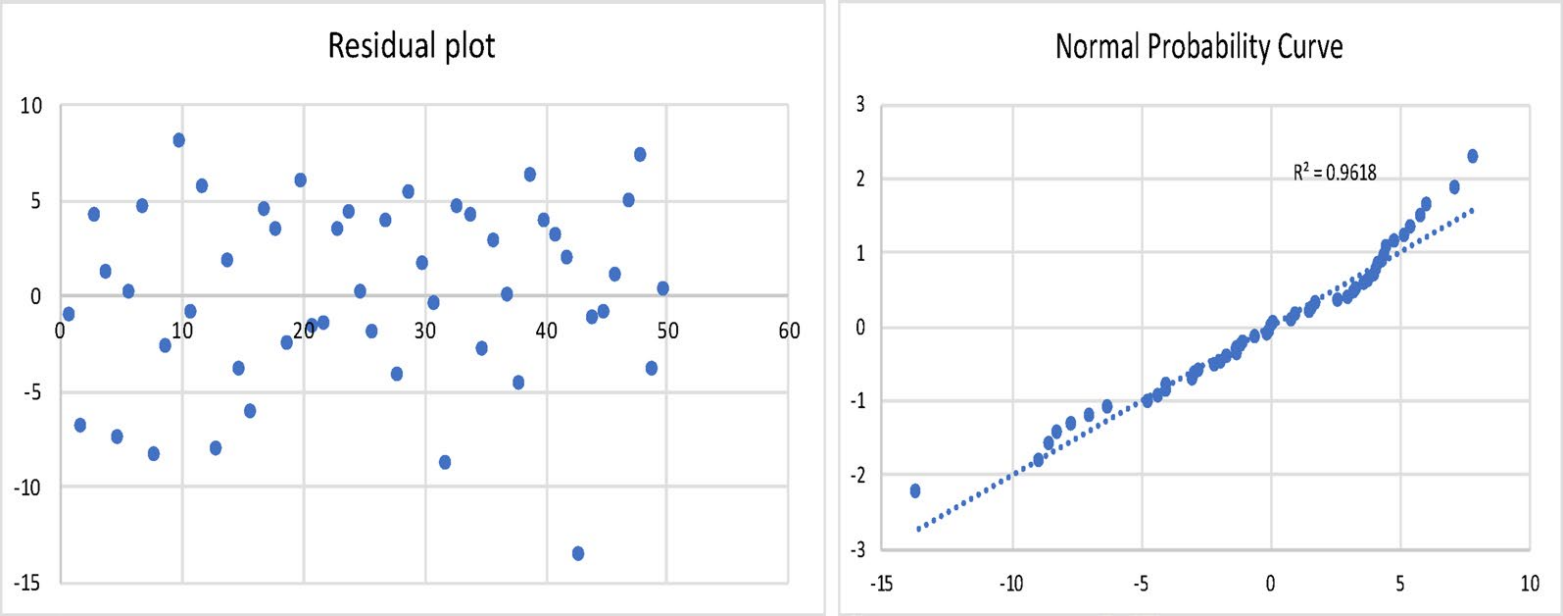
Residual plot and normal probability plot for the gas yield of fast pyrolysis process

The results of the model are shown in Table 2, and the corresponding residual plot and the normal probability curve are shown in Fig. 3*.*

**Table 2:** Regression model results for the gas yield of fast pyrolysis process

	Coefficients	Standard error	*P *value
%Nitrogen (dry basis)	− 5.43	1.01	2.563E-06
%Carbon (dry basis)	− 2.08	0.63	2.008E-03
Temperature (^o^C)	0.05	0.01	1.1093E-10
%Oxygen (dry basis)	− 2.29	0.60	3.959E-04
Free term	215.34	59.05	6.872E-04
*Regression statistics*			
Multiple R	0.91	Adjusted R square	0.80
R square	0.82	Standard error	4.98

All the statistical tests were found to be acceptable. This resulting regression model equation was found to have a minimum error of 0.13%, maximum error of 40%, and an average error of 11%. The Adjusted R Square was found to be 0.8.

4 $$ $$$$ \begin{aligned} Gas~yield~\left( {wt\% } \right) & = - ~5.43 \times \% Nitrogen~dry~basis~ \\ & \quad - ~2.08~ \times \% Carbon~dry~basis \\ & \quad + ~0.05~ \times Temperature~in~~{^\circ } C \\ & \quad - ~2.29 \times ~\% Oxygen~dry~basis \\ ~ & \quad + ~215.34 \\ \end{aligned} $$

As shown, the gas yield in this model depends on temperature, carbon, oxygen, and nitrogen contents. In this model, the temperature increases the gas yield, while oxygen and nitrogen contents decrease the yield. The latter may be attributed to their more significant contribution in the yield of oil. Finally, the carbon content decreases the gas yield as it increases the yield of char.

#### Char yield regression model

**Fig. 4:**
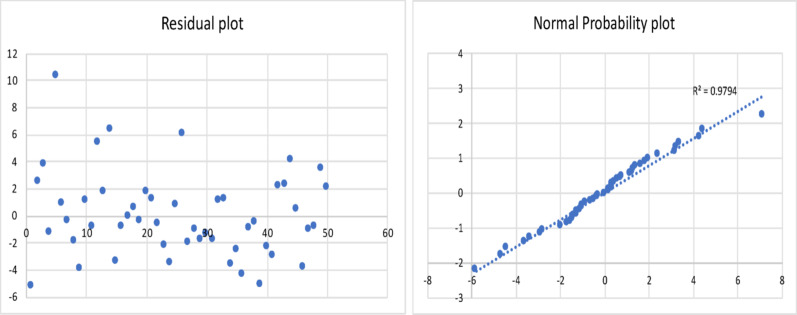
Residual plot and normal probability plot for the char yield of fast pyrolysis process

The results of the model are shown in Table 3, and the corresponding residual plot and the normal probability curve are shown in Fig. 4.

**Table 3:** Regression model results for the char yield of fast pyrolysis process

	Coefficients	Standard error	*P *value
Temperature (^o^C)	− 0.03	0.00	3.0634E-10
Particle size (mm)	2.29	1.13	4.808E-02
%Ash (dry basis)	0.51	0.09	7.8766E-07
Free term	35.60	4.43	3.1209E-10
*Regression statistics*			
Multiple R	0.86	Adjusted R square	0.73
R square	0.75	Standard error	3.26

All the statistical tests were found to be acceptable. This resulting regression model equation was found to have a minimum error of 0.3%, maximum error of 37%, and an average error of 8.6%. The Adjusted R Square was found to be 0.73.

5 $$ $$$$ \begin{aligned} Char~yield~\left( {wt\% } \right) & = ~0.51 \times \% Ash~dry~basis \\ & \quad - ~0.03~ \times ~Temperature~in~{^\circ } C \\ & \quad + ~2.29~ \times ~Particle~size~in~mm~ \\ & \quad + ~35.6 \\ \end{aligned} $$

As shown, the char yield in this model depends on temperature, particle size, and dry ash content. The temperature decreases the char yield, while the ash content and the particle size have the effect of increasing it, which are all logic (Demirbas 2004a, 2004b; Guerrero et al. 2005).

#### Oil yield regression model

**Fig. 5:**
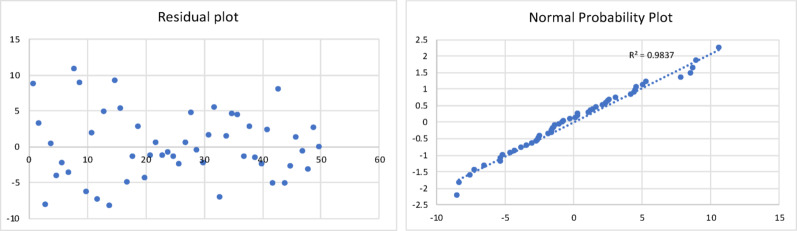
Residualplot and normal probability plot for the oil yield of fast pyrolysis process

The results of the model are shown in Table 4, and the corresponding residual plot and the normal probability curve are shown in Fig. 5.

**Table 4:** Regression model results for the oil yield of fast pyrolysis process

	Coefficients	Standard error	*P *value
%Nitrogen (dry basis)	4.53	0.69	3.826E-08
%Sulfur (dry basis)	− 13.13	3.97	1.87E-03
Temperature (^o^C)	− 0.02	0.01	2.358E-03
Particle size (mm)	− 12.78	1.86	1.5979E-08
Free term	44.61	2.88	1.1819E-19
*Regression statistics*			
Multiple R	0.86	Adjusted R square	0.72
R square	0.74	Standard error	4.88

All the statistical tests were found to be acceptable. The resulting regression model equation was found to have a minimum error of 0.8%, maximum error of 40% and an average error of 15%. The Adjusted R Square was found to be 0.72.

6 $$ \begin{aligned} Oil{\text{ }}yield{\text{ }}\left( {wt\% } \right) & = 4.53 \times \% Nitrogen{\text{ }}dry{\text{ }}basis \\ & \quad - 0.02 \times Temperature{\text{ }}in\,{^\circ } C - 12.7 \\ & \quad \times Particle{\text{ }}size{\text{ }}in{\text{ }}mm - 13.13 \\ & \quad \times \% Sulfur{\text{ }}dry{\text{ }}basis + 44.61 \\ \end{aligned} $$

As shown, the oil yield in this model depends on temperature, particle size, nitrogen and sulfur contents. The temperature decreases the oil yield, which may be attributed to favoring the gas yield (Demirbas 2004a; Şensöz and Can 2002; Pütün 2002; Putun 2004; Şensöz et al. 2006). While the particle size being inversely proportional can be attributed to its role in increasing the char yield (D. Hoornweg PBT 2012). On the other hand, the nitrogen content increases the oil yield, which may be attributed to its contribution to the heavy compounds staying in oil phase (Gómez et al. 2018). Finally, the decrease of the oil yield by increasing sulfur may be attributed to the role of sulfur in increasing the char yield (Zhang et al. 2013; Hu et al. 2014).

### Slow Pyrolysis models

#### Gas yield regression model

**Fig. 6:**
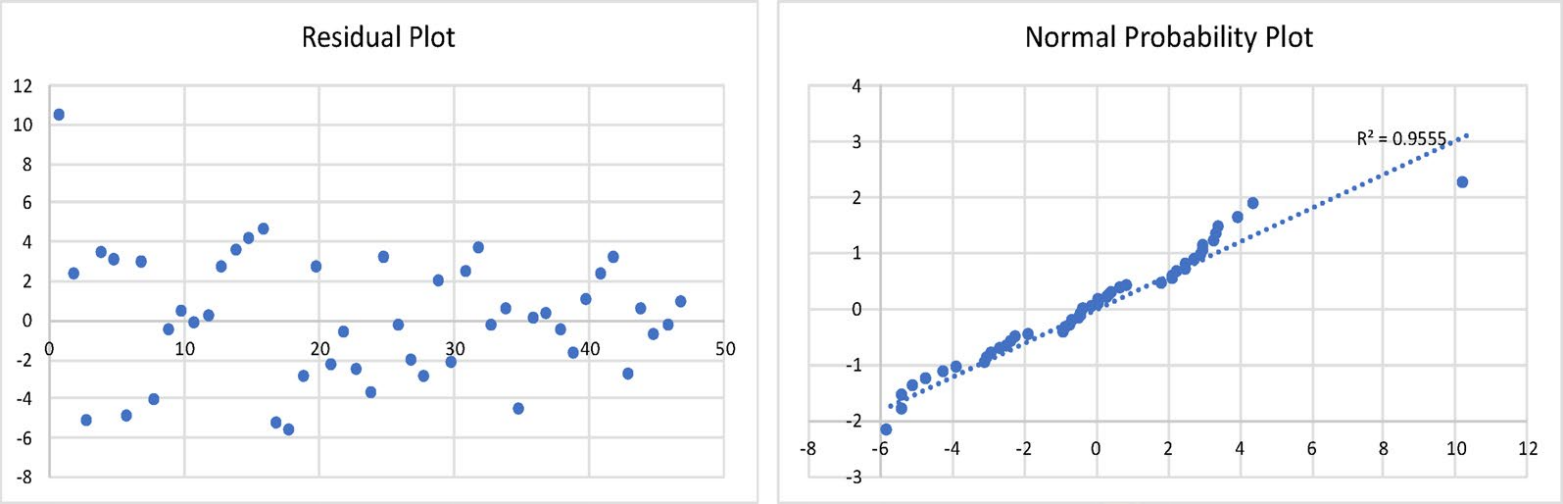
Residual plot and normal probability plot for the gas yield of slow pyrolysis process

The results of the model are shown in Table 5, and the corresponding residual plot and the normal probability curve are shown in Fig. 6.

**Table 5:** Regression model results for the gas yield of slow pyrolysis process

	Coefficients	Standard error	*P *value
%H (dry basis)	− 5.22	1.06	1.52E-05
%S (dry basis)	− 13.38	1.99	4.0291E-08
Temperature	0.04	0.01	1.0135E-05
%C (dry basis)	2.54	0.19	7.751E-17
%N (dry basis)	− 3.67	0.82	5.6319E-05
Free term	− 65.11	8.88	5.6093E-09
*Regression statistics*			
Multiple R	0.95	Adjusted R square	0.89
R Square	0.90	Standard error	3.38

All the statistical tests were found to be acceptable. The resulting regression model equation was found to have a minimum error of 0.01%, maximum error of 19%, and an average error of 4.6%. The Adjusted R Square was found to be 0.89.

7 $$ \begin{aligned} Gas~yield~\left( {wt\% } \right) & = ~0.036\;Temperature~in\,{^\circ } C~ \\ & \quad - 5.22 \times \% Hydrogen~dry~basis~ \\ & \quad - 13.38 \times \% Sulfur~dry~basis + ~2.54 \\ & \quad \times \% Carbon~dry~basis~ - ~3.67 \\ & \quad \times \% Nitrogen~dry~basis~ - ~65.11 \\ \end{aligned} $$

As shown, the gas yield in the second model depends on temperature, hydrogen, sulfur, carbon and nitrogen contents. The temperature increases the yield of gas phase which is according to the literature (Zanzi et al. 1996; Li et al. 2004), while the negative effect of hydrogen, sulfur and nitrogen contents on the gas yield is attributed to their effect on increasing the yield of oil phase or the char. The positive effect of carbon on the gas yield is attributed to the considerable fraction of carbon-containing non-condensable volatiles.

#### Oil yield regression model

**Fig. 7:**
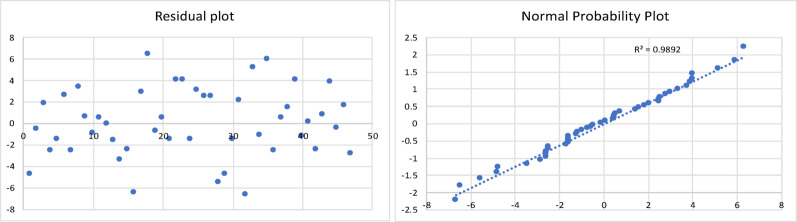
Residual plot and normal probability plot for the oil yield of slow pyrolysis process

The results of the model are shown in Table 6, and the corresponding residual plot and the normal probability curve are shown in Fig. 7.

**Table 6:** Regression model results for the oil yield of slow pyrolysis process

	Coefficients	Standard error	*P *value
%Hydrogen (dry basis)	5.15	1.05	1.59557E-05
%Sulfur (dry basis)	17.53	2.10	2.17522E-10
%Carbon (dry basis)	− 1.35	0.18	5.79811E-09
%Nitrogen (dry basis)	1.95	0.81	0.020265611
Heating rate (^o^C/min)	0.23	0.04	1.50193E-06
Free term	56.54	8.20	2.3093E-08
*Regression statistics*			
Multiple R	0.91	Adjusted R square	0.81
R square	0.83	Standard error	3.34

All the statistical tests were found to be acceptable. The resulting regression model equation was found to have a minimum error of 0.27%, maximum error of 17.3%, and an average error of 6.6%. The Adjusted R Square was found to be 0.81.

8 $$ \begin{aligned} Oil~yield~\left( {wt\% } \right) & = 0.23\;Heating~rate~in~^\circ C\min \\ & \quad + 5.15{\mkern 1mu} \times {\mkern 1mu} \% Hydrogen~dry~basis \\ & \quad + 17.53{\mkern 1mu} \times {\mkern 1mu} \% ~Sulfur~dry~basis \\ & \quad - 1.35{\mkern 1mu} \times {\mkern 1mu} \% Carbon~dry~basis \\ & \quad + 1.95{\mkern 1mu} \times {\mkern 1mu} \% Nitrogen~dry~basis \\ & \quad + ~56.54 \\ \end{aligned} $$

As shown, the oil yield depends on heating rate, hydrogen, sulfur, carbon and nitrogen contents. The heating rate increases the oil yield on the expense of the char yield, while the positive correlation between hydrogen and sulfur contents and the oil yield is attributed to of the nature of the condensable content. The negative effect of the carbon content on the oil yield matches with the interpretation of the gas yield shown above. On the other hand, the positive effect of nitrogen content on the oil yield matches with the interpretation of the gas yield shown above, and it actually needs more investigation.

#### Char yield regression model

**Fig. 8:**
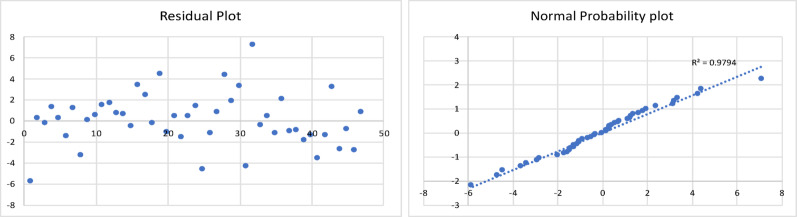
Residual plot and normal probability plot for the char yield of slow pyrolysis process

The results of the model are shown in Table 7, and the corresponding residual plot and the normal probability curve are shown in Fig. 8.

**Table 7:** Regression model results for the char yield of slow pyrolysis process

	Coefficients	Standard error	*P *value
%Oxygen (dry basis)	− 0.24	0.11	2.712E-02
Temperature (^o^C)	− 0.04	0.01	1.5258E-08
%Carbon (dry basis)	− 1.04	0.13	7.1881E-10
Heating rate (^o^ C/min)	− 0.09	0.03	9.575E-03
%FC + Ash (dry basis)	0.28	0.07	3.697E-04
Free term	106.84	9.50	4.1765E-14
*Regression statistics*			
Multiple R	0.89	Adjusted R square	0.77
R square	0.79	Standard error	2.62

All the statistical tests were found to be acceptable. The resulting regression model equation was found to have a minimum error of 0.05%, maximum error of 17%, and an average error of 4.4%. Adjusted R Square was found to be 0.77.

9 $$ \begin{aligned} Char{\text{ }}yield{\text{ }}\left( {wt\% } \right) & = 0.28{\mkern 1mu} \times {\mkern 1mu} \left( {1{\text{ }} - {\text{ }}\% VM{\text{ }}dry{\text{ }}basis} \right) \\ & \quad - {\text{ }}0.24{\mkern 1mu} \times {\mkern 1mu} \% Oxygen{\text{ }}dry{\text{ }}basis \\ & \quad - {\text{ }}0.04{\mkern 1mu} \times {\mkern 1mu} Temperature{\text{ }}in\;^\circ C \\ & \quad - {\text{ }}1.04{\mkern 1mu} \times {\mkern 1mu} \% Carbon{\text{ }}dry{\text{ }}basis \\ & \quad - {\text{ }}0.09{\mkern 1mu} \times {\mkern 1mu} Heating{\text{ }}rate{\text{ }}in^\circ C/\min \\ & \quad + {\text{ }}106.84 \\ \end{aligned} $$

As shown, the char yield depends on temperature, heating rate, carbon content, oxygen content, and dry (FC + Ash). The temperature decreases the char yield which is logic (Couhert et al. 2009), while the negative correlation between carbon content and char yield is attributed to its contribution towards increasing the yield of gas phase as discussed above. The negative effect of oxygen content on char yield can be attributed to its significant contribution to the volatile matter. Finally, the positive effect of the dry (FC + Ash) and negative effect of the heating rate are logic.

### Gasification

#### Methane yield regression model

**Fig. 9:**
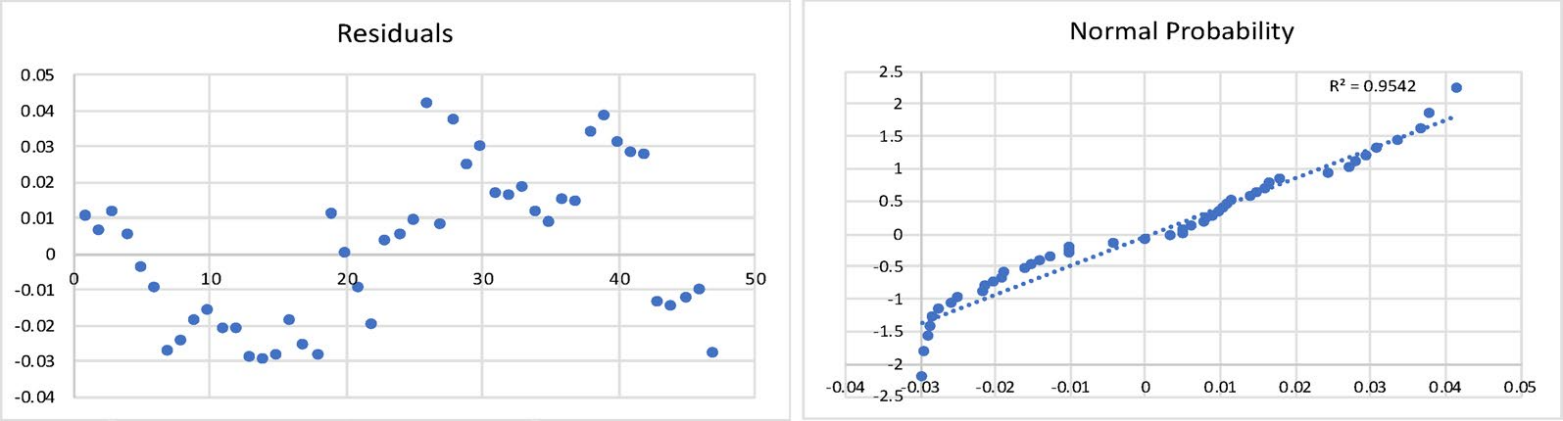
Residual plot and normal probability plot for the methane yield of air gasification process

The results of the model are shown in Table 8, and the corresponding residual plot and the normal probability curve are shown in Fig. 9.

**Table 8:** Regression model results for the methane yield of air gasification process

	Coefficients	Standard error	*P *value
Gasification Temperature (^o^C)	0.0002	0.0001	5.954E-04
%Carbon (dry basis)	− 0.0186	0.0024	8.242E-10
%Hydrogen (dry basis)	0.1131	0.0107	2.0344E-13
%Oxygen (dry basis)	0.0137	0.0014	1.6861E-12
%Moisture	− 0.0083	0.0011	2.6104E-09
Free term	− 0.3205	0.0769	1.512E-04
*Regression statistics*			
Multiple R	0.89	Adjusted R square	0.76
R Square	0.79	Standard error	0.02

All the statistical tests were found to be acceptable. The resulting regression model equation was found to have a minimum error of 0.009%, maximum error of 21.9%, and an average error of 9.8%. The Adjusted R Square was found to be 0.76.

10 $$ \begin{aligned} Methane~yield~\left( {\frac{{m^{3} }}{{kg~feed}}} \right) & = 0.0002{\mkern 1mu} \times {\mkern 1mu} ~Temperature~in~C~ \\ & \quad - 0.0186{\mkern 1mu} \times {\mkern 1mu} \% Carbon~dry~basis \\ & \quad + 0.1131{\mkern 1mu} \times {\mkern 1mu} \% Hydrogen~dry~basis \\ & \quad + 0.0137{\mkern 1mu} \times {\mkern 1mu} \% Oxygen~dry~basis \\ ~ & \quad - ~0.0083{\mkern 1mu} \times {\mkern 1mu} \% Moisture~ - 0.3205 \\ \end{aligned} $$

As shown, the methane yield according to this model equation depends on oxygen, carbon, hydrogen, moisture content and temperature. The positive effect of hydrogen content on methane yield may be attributed to its role in evolving the hydrogen gas as a volatile which then acts as a reactant in methanation reaction (2 CO + 2 H_2_ → CH_4_ + H_2_O) as well as hydrogasification reaction (C + 2 H_2_ → CH_4_). As for the positive effect of the oxygen content, this may be attributed to its role in evolving the CO gas as a volatile which then acts as a reactant in methanation reaction, and may be attributed as well to its role in the generation of hydrogen (via water in the water–gas reaction,^1^ and via water and CO in the water–gas shift reaction^2^) which boosts methane formation. The positive effect of the temperature on methane yield may be attributed to its role in enhancing the kinetics of the methane formation reactions, and also due to its role in driving the endothermic Boudouard (C + CO_2_ → 2CO) and water gas reactions forward and accordingly increasing the CH_4_ precursors (namely CO and H_2_). On the other hand, the negative effect of carbon may be attributed to its bigger role in CO formation. Finally, the negative effect of the moisture content may be attributed to its role in consuming methane in the methane steam reforming (CH_4_ + H_2_O → CO + 3 H_2_).

#### Hydrogen yield regression model

**Fig. 10:**
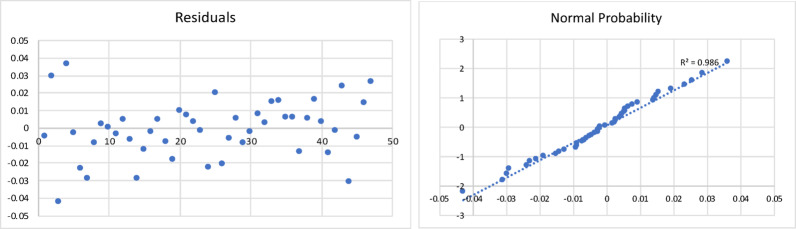
Residual plot and normal probability plot for the hydrogen yield of air gasification process

The results of the model are shown in Table 9, and the corresponding residual plot and the normal probability curve are shown in Fig. 10.

**Table 9:** Regression model results for the hydrogen yield of air gasification process

	Coefficients	Standard Error	*P *value
%Oxygen (dry basis)	0.0076	0.0010	4.5697E-09
%Carbon (dry basis)	− 0.0155	0.0018	3.9957E-11
%Hydrogen (dry basis)	0.0724	0.0092	9.0968E-10
Gasification temperature (^o^C)	0.0002	0.0000	2.822E-05
%VM (dry basis)	− 0.0011	0.0002	6.1226E-07
Free term	0.0517	0.0807	5.253E-01
*Regression statistics*			
Multiple R	0.89	Adjusted R square	0.78
R square	0.80	Standard error	0.02

All the statistical tests were found to be acceptable. The resulting regression model equation was found to have a minimum error of 0.013%, maximum error of 25.17%, and an average error of 7.3%. The Adjusted R Square was found to be 0.78.

11 $$ \begin{aligned} Hydrogen~yield~\left( {\frac{{m^{3} }}{{kg~feed}}} \right) & = ~0.0076{\mkern 1mu} \times {\mkern 1mu} \% Oxygen~dry~basis \\ ~ & \quad - ~0.0155{\mkern 1mu} \times {\mkern 1mu} \% Carbon~dry~basis~ \\ & \quad + ~0.0724{\mkern 1mu} \times {\mkern 1mu} \% Hydrogen~dry~basis~ \\ & \quad + ~0.0002{\mkern 1mu} \times {\mkern 1mu} Temperature~in\,^{^\circ } C~ \\ ~ & \quad - ~0.0011{\mkern 1mu} \times {\mkern 1mu} \% VM~dry~basis \\ & \quad + ~0.0517 \\ \end{aligned} $$

As shown, the hydrogen yield according to this model equation depends on oxygen, carbon, hydrogen, volatile matter and temperature. The positive effect of oxygen and hydrogen contents on increasing the hydrogen yield can be attributed to the role of water in the formation of hydrogen gas via water gas, water gas shift, and steam reformation reactions.

The positive effect of temperature can be attributed to the endothermic nature of the water gas reaction and steam reforming reactions which favor the hydrogen gas formation. On the other hand, the negative effect of carbon may be attributed to its bigger role in CO formation, while the negative effect of the volatile matter can be attributed to its bigger contribution towards the volatile compounds other than hydrogen.

#### Carbon monoxide yield regression model

**Fig. 11:**
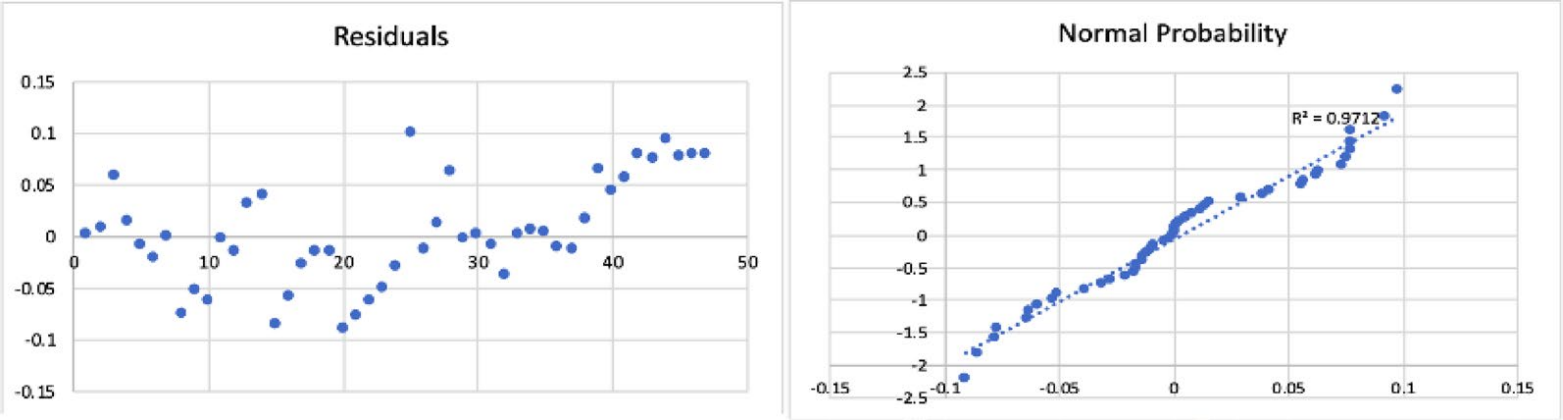
Residual plot and normal probability plot for the carbon monoxide yield of air gasification process

The results of the model are shown in Table 10, and the corresponding residual plot and the normal probability curve are shown in Fig. 11.

**Table 10:** Regression model results for the carbon monoxide yield of air gasification process

	Coefficients	Standard error	*P *value
%Oxygen (dry basis)	0.0122	0.0024	7.9392E-06
%Hydrogen (dry basis)	0.0724	0.0135	2.9776E-06
Gasification temperature (^o^C)	− 0.0002	0.0001	7.343E-03
Free term	− 0.6158	0.1804	1.389E-03
*Regression statistics*			
Multiple R	0.76	Adjusted R square	0.55
R square	0.58	Standard error	0.06

All the statistical tests were found to be acceptable. The resulting regression model equation was found to have a minimum error of 0.006%, maximum error of 30%, and an average error of 11.8%. The Adjusted R Square was found to be 0.55.

12 $$ \begin{aligned} &Carbon~monoxide~yield~\left( {\frac{{m^{3} }}{{kg~feed}}} \right) = ~0.0122 \\ & \qquad \times \% Oxygen~dry~basis \\ & \qquad + ~0.0724{\mkern 1mu} \times {\mkern 1mu} \% Hydrogen~dry~basis \\ ~ & \qquad - ~0.0002{\mkern 1mu} \times {\mkern 1mu} Temperature~in~^{{\text{o}}} C \\ & \qquad - ~0.6158 \\ \end{aligned} $$

As shown, the carbon monoxide yield according to this model equation depends on oxygen, hydrogen, and temperature. The positive effect of oxygen and hydrogen contents on the yield of carbon monoxide is attributed to the role of water in CO formation via water gas and methane steam reforming reactions. On the other hand, the negative effect of temperature may be attributed to the exothermic nature of the partial carbon oxidation reaction which has a main role in CO formation (C + $\frac{1}{2}$ O_2_ → CO).

#### Carbon dioxide yield regression model

**Fig. 12:**
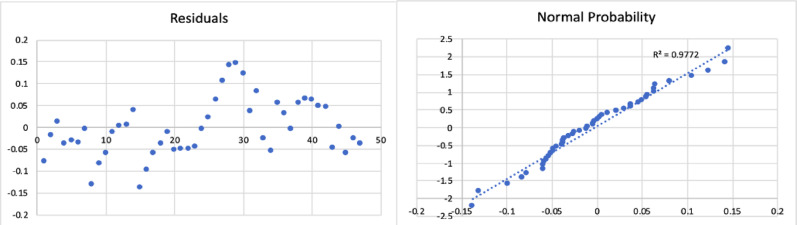
Residual plot and normal probability plot for the carbon dioxide yield of air gasification process

The results of the model are shown in Table 11, and the corresponding residual plot and the normal probability curve are shown in Fig. 12

**Table 11:** Regression model results for the carbon dioxide yield of air gasification process

	Coefficients	Standard error	*P *value
%Carbon (dry basis)	− 0.0272	0.0055	1.2739E-05
%Hydrogen (dry basis)	0.2057	0.0294	1.3048E-08
%Oxygen (dry basis)	0.0216	0.0030	4.6797E-09
%Moisture	− 0.0101	0.0026	3.310E-04
Free term	− 0.4746	0.2235	3.952E-02
*Regression statistics*			
Multiple R	0.78	Adjusted R square	0.57
R square	0.61	Standard error	0.07

All the statistical tests were found to be acceptable. The resulting regression model equation was found to have a minimum error of 0.006%, maximum error of 66%, and an average error of 23.8%. The Adjusted R Square was found to be 0.57.

13 $$ \begin{aligned} &Carbon~dioxide~Yield\left( {\frac{{m^{3} }}{{kg~feed}}} \right) = 0.2057 \\ & \qquad \times \% Hydrogen~dry~basis \\ ~ & \qquad - {\mkern 1mu} 0.0272{\mkern 1mu} ~ \times {\mkern 1mu} \% Carbon~dry~basis \\ & \qquad + {\mkern 1mu} 0.0216{\mkern 1mu} ~ \times {\mkern 1mu} \% Oxygen~dry~basis \\ ~ & \qquad - {\mkern 1mu} 0.0101{\mkern 1mu} ~ \times {\mkern 1mu} \% Moisture - {\mkern 1mu} 0.4746 \\ \end{aligned} $$

As shown, the carbon dioxide yield according to this model depends on hydrogen, carbon, oxygen and moisture. The positive effect of the hydrogen content on the yield of carbon dioxide can be attributed to its presence as part of methane leading to CO_2_ formation via the methane oxidation reaction (CH_4_ + 2 O_2_ → CO_2 +_ 2 H_2_O), and its presence as part of the water leading to CO_2_ formation via the water gas shift reaction. The negative effect of carbon may be attributed to its larger contribution towards carbon monoxide formation. The positive effect of the oxygen content may be attributed to its presence as part of CO leading to CO_2_ formation via the CO oxidation (CO + 0.5 O_2_ → CO_2_) and water gas shift reactions, and its presence as part of the water leading to CO_2_ formation via the water gas shift reactions. The negative effect of moisture may be attributed to its bigger role in CO and H_2_ formation via water gas and methane steam reforming reactions.

To further verify the developed models for each of thermochemical conversion routes, an additional test set of 38 experiments was used utilizing further published experiments in the literature. Upon applying such test sets, the models were found to be acceptable with average error of 17%.

### Utilizing statistical models for feasibility assessment

The developed statistical models presented in the above sections can be used to conduct rapid assessment, from which the yield of products can be predicted from each of the three studied thermochemical processes. Thus, promising techniques can be prioritized rather than the non-promising ones; hence, minimizing the number of required experimental runs.

**Fig. 13:**
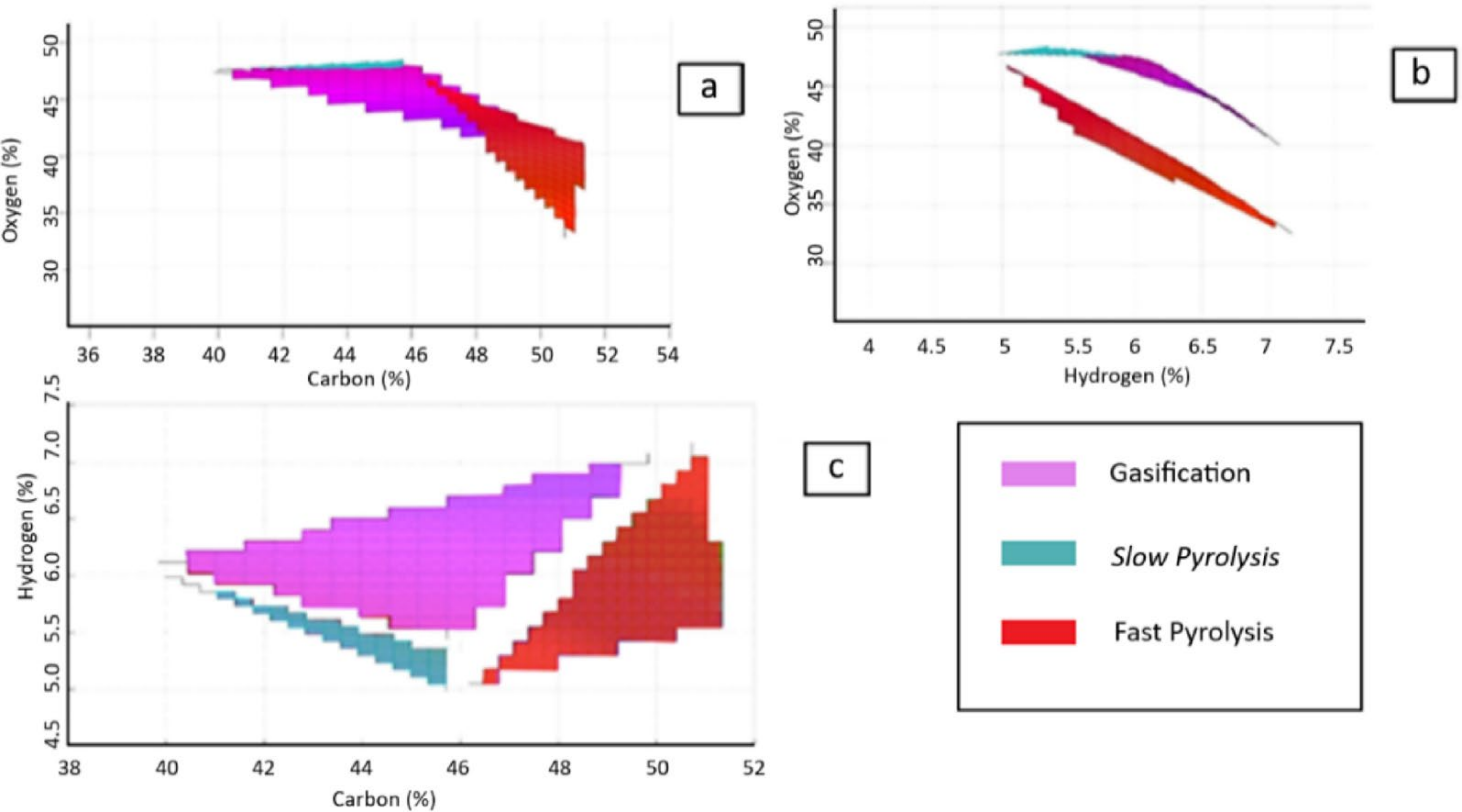
Surface plots of the three studied processes, **a** Oxygen% Vs Carbon%, **b** Oxygen% Versus Hydrogen% and **c** Hydrogen% Versus Carbon%

A decision matrix can be used to evaluate each thermochemical conversion technique on a case-by-case basis. A surface plot was plotted using the accepted values, so an estimation of the accepted range for each of the processes was generated. The surface plots are shown in Fig. 13. From the plots, it was found that:

- If the carbon content lies between 40 and 46%, and the hydrogen content is high, then there is a high probability that gasification is the best technique to deal with the waste.
- If the carbon content is between 40 and 46% and the hydrogen content is low, then there is a high probability that slow pyrolysis is the best technique to deal with the waste.
- If the carbon content is higher than 47%, then there is a high probability that fast pyrolysis is the best technique to deal with the waste.

To verify such conclusions related to the relationship between the biomass elemental analysis and the most promising thermochemical conversion technology, several literature work covering biomass materials used in pilot/demonstration/commercial facilities has been analyzed. One commercial gasifier in China is using fruitwood waste whose ultimate analysis shows that carbon is around 46% and 5.7% hydrogen. Similar observations for biomass compositions were found in other gasification facilities in Japan, China, and Republic of Korea covering biomass materials including municipal waste, tree bark, pine chips, corn stover and cotton stalk (Tanigaki et al. 2012; Fan et al. 2020; Jiang et al. 2023; Pio et al. 2020; Jeong et al. 2020; He et al. 2015; Zhang et al. 2021). For fast pyrolysis, one commercial pyrolyzer in China is using rice husk whose carbon content is 49%. Using biomass materials with carbon content higher than 47% was also found in further fast pyrolysis facilities in Spain, Republic of Ireland, United States of America and India covering biomass materials including pinewood saw dust, forest residues, waste tires, and maize straw (Fernandez-Akarregi et al. 2013; Kwapinska et al. 2021; Martínez et al. 2013; Rathore et al. 2020; Klinger et al. 2020; Cai and Liu 2016). On the other hand, a commercial slow pyrolysis facility in United Kingdom uses Miscanthus whose carbon content is 45% and hydrogen content is about 5%. Similar observations for biomass compositions were found in other slow pyrolysis facilities in United States of America, Italy and Thailand covering biomass materials including corncob, rice straw, sewage sludge and chicken litter (Ro et al. 2010; Brownsort et al. 2011; Salimbeni et al. 2023; vinitnantharat, S., Khaokomol, S., Neamchan, R, Panpradit, B., Sohsalam, P., Werner, D., Mrozik, W. 2021). Such facts match with the above-mentioned conclusions regarding the relation between the ultimate analysis of the biomass and the corresponding most promising thermochemical conversion technology.

## Conclusions

The increasing global focus on sustainable waste management highlights energy conversion as a promising strategy for agricultural and municipal waste. This has led to substantial research in WTE conversion techniques. Recognizing the significant cost and time investments associated with experimental WTE research, this study proposed a supervised machine learning approach by developing statistical models based on published experimental data for various waste types, focusing on bioenergy generation via slow pyrolysis, fast pyrolysis, and gasification.

For each technology, the developed statistical models had a 95% confidence level with low error and correlate the yields of solid, liquid, and gaseous products with waste composition and operating conditions. This provides a guide regarding the expected yield of products for each of the three studied technologies so as to discard the non-promising technologies for the used type of waste and accordingly minimize the number of required experimental runs. Furthermore, a decision matrix was created using these statistical models. This matrix incorporates specific criteria to assess the feasibility of each thermochemical process for a given feedstock. Surface plots generated from this decision matrix indicate that for moderate carbon content (40–46% by mass), gasification is favored when hydrogen content is high; otherwise, slow pyrolysis is preferred. Conversely, fast pyrolysis is favored for waste with high carbon content (above 47%).

## Supplementary Information

Below is the link to the electronic supplementary material.

Supplementary Material 1

Supplementary Material 2

Supplementary Material 3

Supplementary Material 4

## Data Availability

All analyzed data are included in the body of this research article.

## Abbreviations

ANN: Artificial neural networks
BMP: Biochemical methane potential
ER: Equivalence ratio
FC: Fixed carbon
IPCC: Intergovernmental panel on climate change
RF: Random forest
SVM: Support vector machine
VM: Volatile matter
WTE: Waste to energy

## Footnotes

1 C + H_2_O → CO + H_2._

2 CO + H_2_O → CO_2_ + H_2._
