# Supplementary material for: Utilizing machine learning in predicting yields of products in biomass thermochemical conversion processes
Source: Bioresour Bioprocess. 2025 Nov 6;12(1):133. doi: 10.1186/s40643-025-00956-8 (PMC12589710; doi:10.1186/s40643-025-00956-8)
Supplement: Supplementary file 1 — Supplementary Material 1 [file 40643_2025_956_MOESM1_ESM.docx]

Table S-1 Feed type and analysis & operating conditions for slow pyrolysis experiments

| Feed type | O* | C* | N* | H* | S* | Heating rate (^o^C/min) | Particle size (mm) | Temperature (^o^C) | VM* | Ash* | FC* | Gas Yield wt% | Oil Yield wt% | Char Yield wt% |
| --- | --- | --- | --- | --- | --- | --- | --- | --- | --- | --- | --- | --- | --- | --- |
| Rice husk [69] | 40.47 | 48.59 | 2.67 | 8.27 | 0 | 25.00 | 0.45 | 400 | 73.04 | 7.61 | 19.35 | 20.00 | 42.00 | 38.00 |
| Rice husk [69] | 40.47 | 48.59 | 2.67 | 8.27 | 0 | 25.00 | 0.45 | 450 | 73.04 | 7.61 | 19.35 | 22.22 | 45.00 | 33.00 |
| Rice husk [69] | 40.47 | 48.59 | 2.67 | 8.27 | 0 | 25.00 | 0.45 | 500 | 73.04 | 7.61 | 19.35 | 23.00 | 46.00 | 31.00 |
| Rice husk [69] | 40.47 | 48.59 | 2.67 | 8.27 | 0 | 25.00 | 0.45 | 600 | 73.04 | 7.61 | 19.35 | 25.50 | 48.50 | 26.00 |
| Apricot kernel shell [70] | 45.93 | 47.33 | 0.37 | 6.37 | 0 | 10.00 | 0.51 | 500 | 81.79 | 1.04 | 17.18 | 41.00 | 28.00 | 31.00 |
| Apricot kernel shell [70] | 45.93 | 47.33 | 0.37 | 6.37 | 0 | 10.00 | 0.51 | 550 | 81.79 | 1.04 | 17.18 | 44.00 | 26.00 | 30.00 |
| Apricot kernel shell [70] | 45.93 | 47.33 | 0.37 | 6.37 | 0 | 10.00 | 0.51 | 450 | 81.79 | 1.04 | 17.18 | 40.00 | 27.00 | 33.00 |
| Apricot kernel shell [70] | 45.93 | 47.33 | 0.37 | 6.37 | 0 | 10.00 | 0.51 | 400 | 81.79 | 1.04 | 17.18 | 38.00 | 26.00 | 36.00 |
| Apricot kernel shell [70] | 45.93 | 47.33 | 0.37 | 6.37 | 0 | 50.00 | 0.51 | 500 | 81.79 | 1.04 | 17.18 | 33.50 | 39.50 | 27.00 |
| Apricot kernel shell [70] | 45.93 | 47.33 | 0.37 | 6.37 | 0 | 50.00 | 0.51 | 450 | 81.79 | 1.04 | 17.18 | 32.00 | 40.20 | 27.80 |
| Apricot kernel shell [70] | 45.93 | 47.33 | 0.37 | 6.37 | 0 | 50.00 | 0.51 | 400 | 81.79 | 1.04 | 17.18 | 31.00 | 41.00 | 28.00 |
| Grape bagasse [90] | 45.49 | 46.59 | 1.67 | 6.25 | 0 | 10.00 | 0.51 | 350 | 72.94 | 5.01 | 22.05 | 27.00 | 34.00 | 39.00 |
| Grape bagasse [90] | 45.49 | 46.59 | 1.67 | 6.25 | 0 | 10.00 | 0.51 | 400 | 72.94 | 5.01 | 22.05 | 27.00 | 34.00 | 39.00 |
| Grape bagasse [90] | 45.49 | 46.59 | 1.67 | 6.25 | 0 | 10.00 | 0.51 | 450 | 72.94 | 5.01 | 22.05 | 34.00 | 34.50 | 31.50 |
| Grape bagasse [90] | 45.49 | 46.59 | 1.67 | 6.25 | 0 | 10.00 | 0.51 | 500 | 72.94 | 5.01 | 22.05 | 32.00 | 35.50 | 32.50 |
| Grape bagasse [90] | 45.49 | 46.59 | 1.67 | 6.25 | 0 | 10.00 | 0.51 | 550 | 72.94 | 5.01 | 22.05 | 32.00 | 35.50 | 32.50 |
| Grape bagasse [90] | 45.49 | 46.59 | 1.67 | 6.25 | 0 | 10.00 | 0.51 | 600 | 72.94 | 5.01 | 22.05 | 39.00 | 32.00 | 29.00 |
| Grape bagasse [70] | 45.49 | 46.59 | 1.67 | 6.25 | 0 | 50.00 | 0.51 | 450 | 72.94 | 5.01 | 22.05 | 28.00 | 35.00 | 27.00 |
| Grape bagasse [70] | 45.49 | 46.59 | 1.67 | 6.25 | 0 | 50.00 | 0.51 | 500 | 72.94 | 5.01 | 22.05 | 29.00 | 39.00 | 32.00 |
| Grape bagasse [70] | 45.49 | 46.59 | 1.67 | 6.25 | 0 | 50.00 | 0.51 | 600 | 72.94 | 5.01 | 22.05 | 34.00 | 39.00 | 37.00 |
| Olive tree pruning [71] | 42.39 | 50.91 | 0.60 | 6.03 | 0.07 | 15.00 | 0.25 | 450 | 79.67 | 3.73 | 16.60 | 47.99 | 20.05 | 31.96 |
| Olive tree pruning [71] | 42.39 | 50.91 | 0.60 | 6.03 | 0.07 | 15.00 | 0.25 | 550 | 79.67 | 3.73 | 16.60 | 47.47 | 23.24 | 29.30 |
| Pinewood (biomass) [71] | 43.48 | 50.18 | 0.20 | 6.08 | 0.05 | 15.00 | 0.25 | 450 | 83.83 | 0.56 | 15.61 | 48.01 | 26.97 | 25.02 |
| Brunei rice husk [72] | 54.12 | 39.48 | 0.665 | 5.71 | 0.025 | 20 | 0.60 | 450 | 68.25 | 14.83 | 16.92 | 18.47 | 39.61 | 41.92 |
| Brunei rice husk [72] | 54.12 | 39.48 | 0.665 | 5.71 | 0.025 | 21 | 0.60 | 450 | 68.25 | 14.83 | 16.92 | 19.45 | 38.29 | 42.27 |
| Brunei rice husk [72] | 54.12 | 39.48 | 0.665 | 5.71 | 0.025 | 22 | 0.60 | 450 | 68.25 | 14.83 | 16.92 | 18.8 | 39.98 | 43.15 |
| Brunei rice husk [72] | 54.12 | 39.48 | 0.665 | 5.71 | 0.025 | 23 | 0.60 | 450 | 68.25 | 14.83 | 16.92 | 19.18 | 39.59 | 43.31 |
| Brunei rice husk [72] | 54.12 | 39.48 | 0.665 | 5.71 | 0.025 | 24 | 0.60 | 450 | 68.25 | 14.83 | 16.92 | 21.62 | 38.29 | 42.27 |
| Corn Stover [73] | 39.32 | 43.28 | 1.96 | 5.92 | 0.66 | 10.00 | 0.90 | 400 | 82.21 | 8.86 | 8.93 | 15.97 | 42.96 | 41.08 |
| Corn Stover [73] | 39.32 | 43.28 | 1.96 | 5.92 | 0.66 | 10.00 | 0.90 | 500 | 82.21 | 8.86 | 8.93 | 20.23 | 43.92 | 35.86 |
| Corn Stover [73] | 39.32 | 43.28 | 1.96 | 5.92 | 0.66 | 10.00 | 0.90 | 600 | 82.21 | 8.86 | 8.93 | 24.30 | 39.91 | 35.79 |
| Cotton stalk [73] | 41.12 | 43.95 | 1.12 | 5.81 | 0.56 | 10.00 | 0.90 | 400 | 82.38 | 7.45 | 10.17 | 13.93 | 44.36 | 41.72 |
| Cotton stalk [73] | 41.12 | 43.95 | 1.12 | 5.81 | 0.56 | 10.00 | 0.90 | 500 | 82.38 | 7.45 | 10.17 | 17.16 | 47.87 | 34.98 |
| Cotton stalk [73] | 41.12 | 43.95 | 1.12 | 5.81 | 0.56 | 10.00 | 0.90 | 600 | 82.38 | 7.45 | 10.17 | 23.53 | 40.80 | 35.67 |
| Rape stalk [73] | 42.54 | 43.92 | 0.49 | 5.92 | 0.71 | 10.00 | 0.90 | 400-500 | 86.09 | 6.42 | 7.49 | 19.34 | 42.37 | 38.29 |
| Rape stalk [73] | 42.54 | 43.92 | 0.49 | 5.92 | 0.71 | 10.00 | 0.90 | 400-500 | 86.09 | 6.42 | 7.49 | 17.88 | 49.45 | 32.68 |
| Rice straw [73] | 40.23 | 40.06 | 0.69 | 5.47 | 0.48 | 10.00 | 0.90 | 400-600 | 76.87 | 13.07 | 10.06 | 14.72 | 41.56 | 43.73 |
| Rice straw [73] | 40.23 | 40.06 | 0.69 | 5.47 | 0.48 | 10.00 | 0.90 | 500 | 76.87 | 13.07 | 10.06 | 19.64 | 42.87 | 37.5 |
| Rice straw [73] | 40.23 | 40.06 | 0.69 | 5.47 | 0.48 | 10.00 | 0.90 | 600 | 76.87 | 13.07 | 10.06 | 24.11 | 40.31 | 35.59 |
| Wheat straw [73] | 40.51 | 42.95 | 0.76 | 5.64 | 0.78 | 10.00 | 0.90 | 400 | 80.70 | 9.37 | 9.93 | 15.18 | 44.7 | 40.13 |
| Wheat straw [73] | 40.51 | 42.95 | 0.76 | 5.64 | 0.78 | 10.00 | 0.90 | 500 | 80.70 | 9.37 | 9.93 | 19.19 | 46.76 | 34.06 |
| Wheat straw [73] | 40.51 | 42.95 | 0.76 | 5.64 | 0.78 | 10.00 | 0.90 | 600 | 80.70 | 9.37 | 9.93 | 24.04 | 42.33 | 33.64 |
| Pomegranate seeds [74] | 38.13 | 49.65 | 4.03 | 7.54 | 0.65 | 5.00 | 3.20 | 500 | 83.19 | 1.93 | 14.88 | 16.33 | 42.20 | 41.47 |
| Pomegranate seeds [74] | 38.13 | 49.65 | 4.03 | 7.54 | 0.65 | 5.00 | 3.20 | 500 | 83.19 | 1.93 | 14.88 | 16.13 | 45.04 | 29.83 |
| Sugarcane bagasse [74] | 51.00 | 43.00 | 1 | 4.00 | 1.00 | 20.00 | 2.00 | 400 | 90.22 | 3.26 | 6.52 | 18.00 | 44.00 | 38.00 |
| Sugarcane bagasse [74] | 51.00 | 43.00 | 1 | 4.00 | 1.00 | 20.00 | 2.00 | 500 | 90.22 | 3.26 | 6.52 | 25.00 | 47.00 | 28.00 |

*Weight percent (dry basis)

Table S-2 Feed type and analysis & operating conditions for fast pyrolysis experiments

| Feed type | C* | H* | O* | N* | S* | Temperature  (^o^ C) | Particle size  (mm) | Ash* | FC* | VM* | Gas Yield wt% | Oil yield wt% | Char Yield wt% |
| --- | --- | --- | --- | --- | --- | --- | --- | --- | --- | --- | --- | --- | --- |
| Apricot kernel shell [70] | 47.33 | 6.37 | 45.39 | 0.37 | 0 | 400 | 0.51 | 1.04 | 17.18 | 81.79 | 31 | 23 | 28 |
| Apricot kernel shell [70] | 47.33 | 6.37 | 45.39 | 0.37 | 0 | 450 | 0.51 | 1.04 | 17.18 | 81.79 | 32 | 24 | 27.8 |
| Apricot kernel shell [70] | 47.33 | 6.37 | 45.39 | 0.37 | 0 | 500 | 0.51 | 1.04 | 17.18 | 81.79 | 33.5 | 39.5 | 27 |
| Apricot kernel shell [70] | 47.33 | 6.37 | 45.39 | 0.37 | 0 | 550 | 0.51 | 1.04 | 17.18 | 81.79 | 38 | 27 | 35 |
| Grape bagasse [70] | 46.59 | 6.25 | 45.49 | 1.67 | 0 | 400 | 0.51 | 5.01 | 22.05 | 72.94 | 27.50-34.00 | 23.50-39.00 | 27.00-34.00 |
| Grape bagasse [70] | 46.59 | 6.25 | 45.49 | 1.67 | 0 | 450 | 0.51 | 5.01 | 22.05 | 72.94 | 27.5 | 23.5 | 34 |
| Grape bagasse [70] | 46.59 | 6.25 | 45.49 | 1.67 | 0 | 500 | 0.51 | 5.01 | 22.05 | 72.94 | 28 | 24 | 33 |
| Grape bagasse [70] | 46.59 | 6.25 | 45.49 | 1.67 | 0 | 550 | 0.51 | 5.01 | 22.05 | 72.94 | 29 | 39 | 32 |
| Grape bagasse [70] | 46.59 | 6.25 | 45.49 | 1.67 | 0 | 600 | 0.51 | 5.01 | 22.05 | 72.94 | 30 | 40 | 30 |
| Paddy husk [75] | 42.78 | 5.77 | 51.18 | 0.34 | 0 | 400 | 0.43 | 12.16 | 12.16 | 75.67 | 21 | 29 | 50 |
| Paddy husk [75] | 42.78 | 5.77 | 51.18 | 0.34 | 0 | 450 | 0.43 | 12.16 | 12.16 | 75.67 | 23 | 37 | 40 |
| Paddy husk [75] | 42.78 | 5.77 | 51.18 | 0.34 | 0 | 500 | 0.43 | 12.16 | 12.16 | 75.67 | 32 | 30 | 38 |
| Paddy husk [75] | 42.78 | 5.77 | 51.18 | 0.34 | 0 | 550 | 0.43 | 12.16 | 12.16 | 75.67 | 38 | 28 | 34 |
| Paddy husk [75] | 42.78 | 5.77 | 51.18 | 0.34 | 0 | 600 | 0.43 | 12.16 | 12.16 | 75.67 | 39 | 28 | 33 |
| Ceylon tea waste [76] | 58.48 | 6.54 | 31.36 | 3.62 | 0 | 450 | 1.425 | 11.20 | 19.86 | 68.94 | 34 | 28 | 38 |
| Ceylon tea waste [76] | 58.48 | 6.54 | 31.36 | 3.62 | 0 | 500 | 1.425 | 11.20 | 19.86 | 68.94 | 32 | 32 | 36 |
| Ceylon tea waste [76] | 58.48 | 6.54 | 31.36 | 3.62 | 0 | 550 | 1.425 | 11.20 | 19.86 | 68.94 | 35 | 33 | 32 |
| Ceylon tea waste [76] | 58.48 | 6.54 | 31.36 | 3.62 | 0 | 600 | 1.425 | 11.20 | 19.86 | 68.94 | 38 | 33 | 29 |
| Coffee hulls [77] | 47.30 | 6.4 | 37.70 | 2.70 | 0.30 | 500 | 2.00 | 5.60 | 17.40 | 77.00 | 57.20 | 13.57 | 29.23 |
| Coffee hulls [77] | 47.30 | 6.4 | 37.70 | 2.70 | 0.30 | 800 | 2.00 | 5.60 | 17.40 | 77.00 | 62.93 | 11.79 | 25.28 |
| Coffee hulls [77] | 47.30 | 6.4 | 37.70 | 2.70 | 0.30 | 1000 | 2.00 | 5.60 | 17.40 | 77.00 | 64.59 | 11.25 | 24.16 |
| Coffee hulls- microwave assisted [77] | 47.30 | 6.4 | 37.70 | 2.70 | 0.30 | 500 | 2.00 | 5.60 | 17.40 | 77.00 | 61.89 | 7.90 | 30.21 |
| Coffee hulls- microwave assisted [77] | 47.30 | 6.4 | 37.70 | 2.70 | 0.30 | 800 | 2.00 | 5.60 | 17.40 | 77.00 | 65.28 | 9.19 | 25.53 |
| Coffee hulls- microwave assisted [77] | 47.30 | 6.4 | 37.70 | 2.70 | 0.30 | 1000 | 2.00 | 5.60 | 17.40 | 77.00 | 68.72 | 8.58 | 22.70 |
| Camellia seed shell [78] | 46.05 | 6.08 | 47.33 | 0.37 | 0.17 | 400 | 0.08 | 4.47 | 22.20 | 73.33 | 34.56 | 27.32 | 38.12 |
| Camellia seed shell [78] | 46.05 | 6.08 | 47.33 | 0.37 | 0.17 | 500 | 0.08 | 4.47 | 22.20 | 73.33 | 40.03 | 28.63 | 31.34 |
| Camellia seed shell [78] | 46.05 | 6.08 | 47.33 | 0.37 | 0.17 | 600 | 0.08 | 4.47 | 22.20 | 73.33 | 45.46 | 24.78 | 29.76 |
| Camellia seed shell [78] | 46.05 | 6.08 | 47.33 | 0.37 | 0.17 | 800 | 0.08 | 4.47 | 22.20 | 73.33 | 50.1 | 22.92 | 26.98 |
| Rapeseed straw [78] | 44.39 | 6.47 | 48.24 | 0.54 | 0.36 | 400 | 0.08 | 5.42 | 18.20 | 76.38 | 35.36 | 30.16 | 34.48 |
| Rapeseed straw [78] | 44.39 | 6.47 | 48.24 | 0.54 | 0.36 | 500 | 0.08 | 5.42 | 18.20 | 76.38 | 39.45 | 30.73 | 29.82 |
| Rapeseed straw [78] | 44.39 | 6.47 | 48.24 | 0.54 | 0.36 | 600 | 0.08 | 5.42 | 18.20 | 76.38 | 47.41 | 28.45 | 24.14 |
| Rapeseed straw [78] | 44.39 | 6.47 | 48.24 | 0.54 | 0.36 | 800 | 0.08 | 5.42 | 18.20 | 76.38 | 52.69 | 27.64 | 19.67 |
| Camellia seed meal [78] | 50.64 | 7.12 | 40.77 | 1.16 | 0.31 | 400 | 0.08 | 2.73 | 19.83 | 77.44 | 24.7 | 40.47 | 34.83 |
| Camellia seed meal [78] | 50.64 | 7.12 | 40.77 | 1.16 | 0.31 | 500 | 0.08 | 2.73 | 19.83 | 77.44 | 30.63 | 40.66 | 28.71 |
| Camellia seed meal [78] | 50.64 | 7.12 | 40.77 | 1.16 | 0.31 | 600 | 0.08 | 2.73 | 19.83 | 77.44 | 33.18 | 39.02 | 27.8 |
| Camellia seed meal [78] | 50.64 | 7.12 | 40.77 | 1.16 | 0.31 | 800 | 0.08 | 2.73 | 19.83 | 77.44 | 38.91 | 37.83 | 23.26 |
| Rapeseed meal [78] | 48.62 | 7.45 | 37.46 | 5.50 | 0.97 | 400 | 0.08 | 5.52 | 12.35 | 82.13 | 19.88 | 45.96 | 34.16 |
| Rapeseed meal [78] | 48.62 | 7.45 | 37.46 | 5.50 | 0.97 | 500 | 0.08 | 5.52 | 12.35 | 82.13 | 23.35 | 46.89 | 29.76 |
| Rapeseed meal [78] | 48.62 | 7.45 | 37.46 | 5.50 | 0.97 | 600 | 0.08 | 5.52 | 12.35 | 82.13 | 25.54 | 47.37 | 27.09 |
| Rapeseed meal [78] | 48.62 | 7.45 | 37.46 | 5.50 | 0.97 | 800 | 0.08 | 5.52 | 12.35 | 82.13 | 39.7 | 38.02 | 22.28 |
| Rice husk [79] | 38.10 | 5.00 | 56.10 | 0.26 | 0.52 | 400 | 0.45 | 23.57 | 20.18 | 56.25 | 19 | 36.5 | 44.5 |
| Rice husk [79] | 38.10 | 5.00 | 56.10 | 0.26 | 0.52 | 500 | 0.45 | 23.57 | 20.18 | 56.25 | 37 | 23 | 40 |
| Rice husk [79] | 38.10 | 5.00 | 56.10 | 0.26 | 0.52 | 700 | 0.45 | 23.57 | 20.18 | 56.25 | 45 | 15 | 40 |
| Rubber wood dust [80] | 46.60 | 7.10 | 46.00 | 0.30 | 0 | 450 | 0.33 | 1.35 | 16.04 | 82.60 | 37 | 25 | 38 |
| Rubber wood dust [80] | 46.60 | 7.10 | 46.00 | 0.30 | 0 | 500 | 0.33 | 1.35 | 16.04 | 82.60 | 38 | 31 | 31 |
| Rubber wood dust [80] | 46.60 | 7.10 | 46.00 | 0.30 | 0 | 550 | 0.33 | 1.35 | 16.04 | 82.60 | 40 | 33 | 27 |
| Rubber wood dust [80] | 46.60 | 7.10 | 46.00 | 0.30 | 0 | 600 | 0.33 | 1.35 | 16.04 | 82.60 | 47 | 28 | 25 |
| Meranti wood sawdust [80] | 41.70 | 5.70 | 52.60 | 0.10 | 0 | 450 | 0.33 | 1.28 | 24.20 | 74.52 | 37 | 25 | 38 |
| Meranti wood sawdust [80] | 41.70 | 5.70 | 52.60 | 0.10 | 0 | 500 | 0.33 | 1.28 | 24.20 | 74.52 | 40 | 27 | 33 |
| Meranti wood sawdust [80] | 41.70 | 5.70 | 52.60 | 0.10 | 0 | 550 | 0.33 | 1.28 | 24.20 | 74.52 | 42 | 30 | 28 |
| Meranti wood sawdust [80] | 41.70 | 5.70 | 52.60 | 0.10 | 0 | 600 | 0.33 | 1.28 | 24.20 | 74.52 | 42 | 34 | 24 |
| Meranti wood sawdust [80] | 41.70 | 5.70 | 52.60 | 0.10 | 0 | 650 | 0.33 | 1.28 | 24.20 | 74.52 | 45 | 31 | 24 |

*Weight percent (dry basis)

Table S-3 Feed type and analysis & operating conditions for gasification experiments

| Feed Type | N* | C* | H* | O* | S* | Temperature  (^o^C) | ER | VM dry | FC dry | Ash dry | H_2_ yield m^3^ | CO yield | CO_2_ yield | CH_4_ yield |
| --- | --- | --- | --- | --- | --- | --- | --- | --- | --- | --- | --- | --- | --- | --- |
| Solid recovered fuel [82] | 0.92 | 58.82 | 8.40 | 31.58 | 0.27 | 849 | 0.33 | 0.00 | 0.00 | 100.0 | 0.14 | 0.16 | 0.22 | 0.07 |
| Solid recovered fuel [82] | 0.92 | 58.82 | 8.40 | 31.58 | 0.27 | 852 | 0.33 | 0.00 | 0.00 | 100.0 | 0.12 | 0.16 | 0.27 | 0.07 |
| Solid recovered fuel [82] | 0.92 | 58.82 | 8.40 | 31.58 | 0.27 | 869 | 0.33 | 0.00 | 0.00 | 100.0 | 0.15 | 0.18 | 0.21 | 0.08 |
| Solid recovered fuel [82] | 0.92 | 58.82 | 8.40 | 31.58 | 0.27 | 879 | 0.33 | 0.00 | 0.00 | 100.0 | 0.16 | 0.01 | 0.25 | 0.08 |
| Solid recovered fuel [82] | 0.92 | 58.82 | 8.40 | 31.58 | 0.27 | 932 | 0.33 | 0.00 | 0.00 | 100.0 | 0.15 | 0.21 | 0.23 | 0.08 |
| Municipal solid waste [79] | 2.90 | 46.27 | 5.20 | 44.77 | 0.86 | 760 | 0.34 | 46.95 | 8.49 | 44.56 | 0.12 | 0.15 | 0.16 | 0.03 |
| Municipal solid waste [79] | 2.90 | 46.27 | 5.20 | 44.77 | 0.86 | 800 | 0.42 | 46.95 | 8.49 | 44.56 | 0.11 | 0.15 | 0.2 | 0.03 |
| Meat and bone meal [83] | 9.10 | 54.71 | 7.80 | 27.61 | 0.78 | 800 | 0.25 | 71.88 | 9.07 | 19.04 | 0.07 | 0.1 | 0.13 | 0.04 |
| Meat and bone meal [83] | 9.10 | 54.71 | 7.80 | 27.61 | 0.78 | 820 | 0.29 | 71.88 | 9.07 | 19.04 | 0.04 | 0.07 | 0.14 | 0.04 |
| Meat and bone meal [83] | 9.10 | 54.71 | 7.80 | 27.61 | 0.78 | 830 | 0.31 | 71.88 | 9.07 | 19.04 | 0.02 | 0.06 | 0.13 | 0.03 |
| Meat and bone meal [83] | 9.10 | 54.71 | 7.80 | 27.61 | 0.78 | 845 | 0.42 | 71.88 | 9.07 | 19.04 | 0.02 | 0.08 | 0.17 | 0.03 |
| Municipal solid waste [83] | 1.56 | 45.87 | 4.92 | 47.7 | 0 | 450 | 0.15 | 82.10 | 14.40 | 4.50 | 0.04 | 0.14 | 0.11 | 0.04 |
| Municipal solid waste [83] | 1.56 | 45.87 | 4.92 | 47.7 | 0 | 450 | 0.2 | 82.10 | 14.40 | 4.50 | 0.05 | 0.16 | 0.16 | 0.04 |
| Municipal solid waste [83] | 1.56 | 45.87 | 4.92 | 47.7 | 0 | 450 | 0.25 | 82.10 | 14.40 | 4.50 | 0.05 | 0.15 | 0.18 | 0.05 |
| Municipal solid waste [83] | 1.56 | 45.87 | 4.92 | 47.7 | 0 | 450 | 0.3 | 82.10 | 14.40 | 4.50 | 0.04 | 0.21 | 0.23 | 0.05 |
| Municipal solid waste [83] | 1.56 | 45.87 | 4.92 | 47.7 | 0 | 450 | 0.35 | 82.10 | 14.40 | 4.50 | 0.05 | 0.2 | 0.25 | 0.04 |
| Municipal solid waste [84] | 1.56 | 45.85 | 4.92 | 47.68 | 0.00 | 450 | 0.15 | 80.91 | 14.26 | 4.83 | 0.04 | 0.13 | 0.11 | 0.04 |
| Municipal solid waste [84] | 1.56 | 45.85 | 4.92 | 47.68 | 0.00 | 450 | 0.2 | 80.91 | 14.26 | 4.83 | 0.05 | 0.15 | 0.14 | 0.04 |
| Municipal solid waste [84] | 1.56 | 45.85 | 4.92 | 47.68 | 0.00 | 450 | 0.25 | 80.91 | 14.26 | 4.83 | 0.05 | 0.18 | 0.18 | 0.05 |
| Municipal solid waste [84] | 1.56 | 45.85 | 4.92 | 47.68 | 0.00 | 450 | 0.3 | 80.91 | 14.26 | 4.83 | 0.04 | 0.2 | 0.21 | 0.04 |
| Municipal solid waste [84] | 1.56 | 45.85 | 4.92 | 47.68 | 0.00 | 450 | 0.35 | 80.91 | 14.26 | 4.83 | 0.03 | 0.2 | 0.23 | 0.04 |
| Palm kernel shell [85] | 1.89 | 51.63 | 5.52 | 40.91 | 0.05 | 750 | 0.15 | 72.47 | 18.56 | 8.97 | 0.02 | 0.01 | 0.01 | 0 |
| Palm kernel shell [85] | 1.89 | 51.63 | 5.52 | 40.91 | 0.05 | 800 | 0.15 | 72.47 | 18.56 | 8.97 | 0.03 | 0.01 | 0.01 | 0 |
| Palm kernel shell [85] | 1.89 | 51.63 | 5.52 | 40.91 | 0.05 | 850 | 0.15 | 72.47 | 18.56 | 8.97 | 0.03 | 0.01 | 0.01 | 0 |
| Palm kernel shell [85] | 1.89 | 51.63 | 5.52 | 40.91 | 0.05 | 900 | 0.15 | 72.47 | 18.56 | 8.97 | 0.03 | 0.01 | 0.02 | 0 |
| Poultry litter [86] | 3.90 | 42.82 | 5.49 | 32.69 | 0.60 | 700 | 0.17 | 67.37 | 20.13 | 12.50 | 0.07 | 0.05 | 0.09 | 0.02 |
| Poultry litter [86] | 3.90 | 42.82 | 5.49 | 32.69 | 0.60 | 700 | 0.17 | 67.37 | 20.13 | 12.50 | 0.08 | 0.06 | 0.12 | 0.02 |
| Poultry litter [86] | 3.90 | 42.82 | 5.49 | 32.69 | 0.60 | 700 | 0.17 | 67.37 | 20.13 | 12.50 | 0.1 | 0.09 | 0.16 | 0.02 |
| Rice straw [87] | 1.46 | 52.13 | 6.27 | 39.91 | 0.24 | 600 | 0.2 | 64.73 | 20.36 | 14.92 | 0.01 | 0.15 | 0.34 | 0.06 |
| Rice straw [87] | 1.46 | 52.13 | 6.27 | 39.91 | 0.24 | 800 | 0.2 | 64.73 | 20.36 | 14.92 | 0.09 | 0.23 | 0.28 | 0.07 |
| Rice straw [87] | 1.46 | 52.13 | 6.27 | 39.91 | 0.24 | 650 | 0.2 | 64.73 | 20.36 | 14.92 | 0.02 | 0.15 | 0.32 | 0.07 |
| Rice straw [87] | 1.46 | 52.13 | 6.27 | 39.91 | 0.24 | 700 | 0.2 | 64.73 | 20.36 | 14.92 | 0.04 | 0.17 | 0.32 | 0.07 |
| Rice straw [87] | 1.46 | 52.13 | 6.27 | 39.91 | 0.24 | 750 | 0.2 | 64.73 | 20.36 | 14.92 | 0.06 | 0.2 | 0.3 | 0.08 |
| Rice straw [87] | 1.46 | 52.13 | 6.27 | 39.91 | 0.24 | 700 | 0.15 | 64.73 | 20.36 | 14.92 | 0.04 | 0.15 | 0.22 | 0.06 |
| Rice straw [87] | 1.46 | 52.13 | 6.27 | 39.91 | 0.24 | 700 | 0.17 | 64.73 | 20.36 | 14.92 | 0.05 | 0.15 | 0.26 | 0.06 |
| Saw dust 1 [88] | 0.81 | 49.15 | 5.74 | 44.31 | 0.00 | 700 | 0.2 | 83.02 | 15.90 | 1.08 | 0.08 | 0.16 | 0.12 | 0.03 |
| Saw dust 1 [88] | 0.81 | 49.15 | 5.74 | 44.31 | 0.00 | 700 | 0.15 | 83.02 | 15.90 | 1.08 | 0.07 | 0.13 | 0.09 | 0.02 |
| Saw dust 1 [88] | 0.81 | 49.15 | 5.74 | 44.31 | 0.00 | 700 | 0.35 | 83.02 | 15.90 | 1.08 | 0.09 | 0.17 | 0.2 | 0.02 |
| Saw dust 1 [88] | 0.81 | 49.15 | 5.74 | 44.31 | 0.00 | 700 | 0.3 | 83.02 | 15.90 | 1.08 | 0.09 | 0.17 | 0.18 | 0.03 |
| Saw dust 1 [88] | 0.81 | 49.15 | 5.74 | 44.31 | 0.00 | 700 | 0.25 | 83.02 | 15.90 | 1.08 | 0.08 | 0.17 | 0.14 | 0.03 |
| Saw dust 2 [88] | 0.10 | 46.07 | 5.34 | 48.47 | 0.02 | 500 | 0.2 | 76.77 | 17.61 | 0.40 | 0.06 | 0.31 | 0.42 | 0.18 |
| Saw dust 2 [88] | 0.10 | 46.07 | 5.34 | 48.47 | 0.02 | 500 | 0.22 | 76.77 | 17.61 | 0.40 | 0.09 | 0.33 | 0.43 | 0.19 |
| Saw dust 2 [88] | 0.10 | 46.07 | 5.34 | 48.47 | 0.02 | 500 | 0.24 | 76.77 | 17.61 | 0.40 | 0.11 | 0.32 | 0.43 | 0.18 |
| Saw dust 2 [88] | 0.10 | 46.07 | 5.34 | 48.47 | 0.02 | 500 | 0.26 | 76.77 | 17.61 | 0.40 | 0.12 | 0.32 | 0.41 | 0.18 |
| Saw dust 2 [88] | 0.10 | 46.07 | 5.34 | 48.47 | 0.02 | 500 | 0.28 | 76.77 | 17.61 | 0.40 | 0.16 | 0.32 | 0.41 | 0.18 |

*Weight percent (dry basis)
