# Supplementary material for: Utilizing machine learning in predicting yields of products in biomass thermochemical conversion processes
Source: Bioresour Bioprocess. 2025 Nov 6;12(1):133. doi: 10.1186/s40643-025-00956-8 (PMC12589710; doi:10.1186/s40643-025-00956-8)
Supplement: Supplementary file 3 — Supplementary Material 3 [file 40643_2025_956_MOESM3_ESM.docx]

**Name**: Ahmad Wafiq

**Phone**: +201003704390

**E-mail Address**: ahmad.wafiq@eng1.cu.edu.eg

**Address**: Chemical Engineering
Department, Faculty of Engineering, Cairo University, Egypt
**Date**: September 23^rd^ 2025

Dear editorial board of “Bioresources and Bioprocessing”,

We would like to thank you for giving us the chance to submit a revised manuscript. Please find enclosed the revised manuscript titled *"* **Utilizing Machine Learning in Predicting Yields of Products in Biomass Thermochemical Conversion Processes***".* We have carefully addressed all comments from the reviewers, resulting in significant improvements to the manuscript. The revised manuscript (in track changes) is uploaded together with a document including the responses to the reviewers’ comments, and the revised supplementary tables.

We believe these enhancements have strengthened the manuscript and aligned it with the journal’s high standards. We sincerely thank the editor and the reviewers for their constructive feedback.

We look forward to your positive consideration.

Sincerely,
Ahmad Wafiq

(On behalf of all authors)
