# Supplementary figures and images for: Utilizing machine learning in predicting yields of products in biomass thermochemical conversion processes
Source: Bioresour Bioprocess. 2025 Nov 6;12(1):133. doi: 10.1186/s40643-025-00956-8 (PMC12589710; doi:10.1186/s40643-025-00956-8)

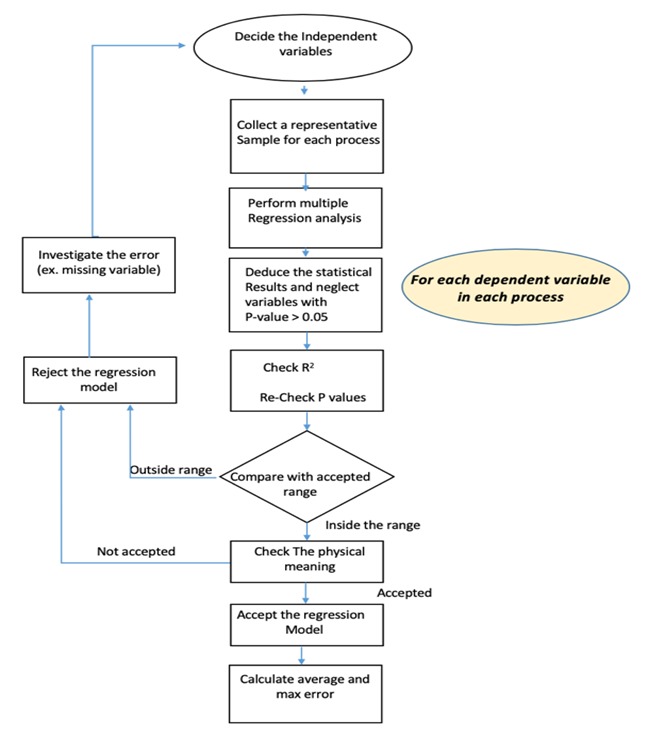

Supplement: Supplementary file 4 — Supplementary Material 4 [file 40643_2025_956_MOESM4_ESM.jpg]
