# Supplementary material for: Utilizing machine learning in predicting yields of products in biomass thermochemical conversion processes
Source: Bioresour Bioprocess. 2025 Nov 6;12(1):133. doi: 10.1186/s40643-025-00956-8 (PMC12589710; doi:10.1186/s40643-025-00956-8)
Supplement: Supplementary file 2 — Supplementary Material 2 [file 40643_2025_956_MOESM2_ESM.docx]

Dear Editor,

Thank you for your time and your valuable assistance. All the comments have been taken into consideration in the modified manuscript as shown in the following responses. We hope the revised manuscript meets your expectations.

**Reviewer (1):**

Thank you for your time and your valuable comments. All the comments have been taken into consideration as shown below

1.On page 5, in the method section, the author mentions that this paper employs multiple linear regression and explains that this method can only be used for linear relationships. Therefore, the author should explain why this model was chosen instead of others, as there are many models that can fit nonlinear relationships or the author could provide the reasons why the research conducted in this paper can only use linear relationship fitting.

Thanks for your valuable comment. The “Applied statistical modeling techniques” section has been enriched to address your comment.

Previously, it was suggested that the author add descriptions of other regression models. Is the only regression model used by the author linear regression? If only the multiple linear regression is used, then please explain, based on the first comment and the review comments of the second reviewer, why only this model is used and other models are not considered.

Thanks for your valuable comment. The “Applied statistical modeling techniques” section has been enriched to address your comment.
